# Controlled Synthesis of a New Class of Heterostructured Metal Oxides (Cerium, Thorium, Uranium)/Calcium Fluoride Core‐Shell Nanocrystals With Atomically Coherent Interfaces

**DOI:** 10.1002/anie.202524282

**Published:** 2026-02-15

**Authors:** Dejing Meng, Radian Popescu, Carmen M. Andrei, Jérôme Himbert, Eduard Madirov, Jacob A. Branson, Emily M. Reynolds, Tim Prüßmann, Jörg Göttlicher, Tonya Vitova, Niko Hildebrandt, Yolita Eggeler, Bryce S. Richards, Olaf Walter, Damien Hudry

**Affiliations:** ^1^ Institute of Microstructure Technology Karlsruhe Institute of Technology Karlsruhe Germany; ^2^ Laboratory For Electron Microscopy Karlsruhe Institute of Technology Karlsruhe Germany; ^3^ Canadian Center for Electron Microscopy McMaster University Hamilton Ontario Canada; ^4^ European Commission, Joint Research Centre (JRC) Karlsruhe Germany; ^5^ Department of Engineering Physics McMaster University Hamilton Ontario Canada; ^6^ Institute For Nuclear Waste Disposal Karlsruhe Institute of Technology Karlsruhe Germany; ^7^ Institute For Photon Science and Synchrotron Radiation Karlsruhe Institute of Technology Karlsruhe Germany; ^8^ Light Technology Institute Karlsruhe Institute of Technology Karlsruhe Germany

**Keywords:** actinides, calcium fluoride, core‐shell nanocrystals, heterostructure, metal oxide

## Abstract

Heterostructured nanocrystals (NCs) integrating chemically and/or structurally distinct materials are of significant interest due to their potential to exhibit multiple functionalities or unconventional properties. However, the development of such materials remains limited, primarily due to crystallographic incompatibilities and synthesis challenges. Here, we report the synthesis and structural characterization of a new class of metal oxide‐based (Ce, Th, or U) heterostructures with calcium fluoride featuring core‐shell architectures with precise control over both morphology and shell thickness. Powder x‐ray diffraction (PXRD) and high‐resolution scanning/transmission electron microscopy techniques confirm the formation of high‐quality single‐crystalline particles coupling metal oxide (Ce, Th, or U) and calcium fluoride domains through a structurally coherent but chemically complex intermixed oxide‐fluoride interface—an unprecedented observation in cerium and actinide nanochemistry. These novel materials have been designed to anticipate their future doping with therapeutic alpha particle‐emitting radionuclides (α‐emitters) such as ^227^Th or ^230^U for locoregional targeted alpha therapy (TAT). It is anticipated that the reported heterostructured core‐shell NCs will offer a promising platform for preclinical investigations to determine the full potential and interest of inorganic core‐shell NCs, especially CeO_2 _/ CaF_2_, for TAT application.

## Graphical abstract

Precisely engineered heterostructured metal oxide‐based MO_2 _/ CaF_2_ (M = Ce, Th, U) core‐shell nanocrystals are synthesized with atomic‐level control over size, shell thickness, and interface coherence, expanding heterostructure design principles to cerium oxide and actinide oxide systems for the first time.

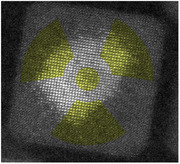

The controlled synthesis of heterogeneous nanocrystals (NCs) made of at least two disparate materials that are precisely integrated to form a well‐defined architecture creates a pathway to access multiple functionalities and even unconventional properties due to the synergetic coupling between individual nano‐sized domains [1]. Among those architectures, core‐shell nanostructures represent an important class of nanomaterials, which are of major interest for a wide range of technological applications [2].

Targeted alpha therapy (TAT) is an emerging and powerful modality in nuclear medicine that leverages the short penetration range and high linear energy transfer of therapeutic alpha particle‐emitting radionuclides (α‐emitters) to revolutionize cancer care [3, 4, 5]. Although the field is widely dominated by fully organic radiopharmaceuticals [6], the past decade has witnessed the emergence of various inorganic delivery platforms such as core‐shell NCs [7, 8]. Unfortunately, core‐shell structures investigated for TAT and reported in the literature suffer from several limitations, including (i) poorly controlled size and shape distributions; (ii) uncontrolled (if any) shell thickness; (iii) lack of a structurally and/or chemically coherent interface between the core and shell domains; and (iv) significant interparticle agglomeration issues [9, 10, 11, 12, 13, 14, 15]. Considering the critical role these parameters play—not only to prevent the uncontrolled release of radiotoxic recoiled daughters but also on the in vivo behavior of core‐shell NCs [16, 17, 18], model systems are urgently needed to acquire highly reliable preclinical data to properly assess the true interest and performance of these materials for TAT while providing a realistic assessment regarding their potential in terms of clinical translation. Although such a model system has been recently reported for radionuclide therapy with β‐emitters (^177^Lu) [19], there are currently no reasonable lead candidates for TAT application.

In this work, we hypothesize that MO_2_ metal oxides (M = Ce, Th, U) can be combined with CaF_2_ to form an unprecedented class of heterostructured core‐shell NCs due to isomorphism and limited lattice mismatch (Figures 1a–c). The CeO_2_/CaF_2_ heterostructure is designed as a structurally and chemically compatible host matrix to anticipate its direct doping with the therapeutic α‐emitter ^227^Th and thus, its ability to act as an in vivo generator for ^223^Ra (Figure 1d). The CeO_2 _/ CaF_2_ core‐shell NCs might reveal as promising candidates for TAT due to important features including (i) biocompatibility [17, 18, 20, 21], (ii) chemical and structural stability of cubic metal oxides under extreme conditions [22, 23, 24, 25], and (iii) chemical biocompatibility of the CaF_2_ shell domain with both long‐lived radionuclides (^223^Ra and ^211^Pb) and the final stable decay element (^207^Pb) [26], which are generated during the complex radioactive decay cascade of ^227^Th (Figure 1d). To evaluate the generality of this approach and demonstrate the validity of the concept for TAT, the first AnO_2_‐based core‐shell NCs (i.e., ThO_2 _/ CaF_2_ and UO_2 _/ CaF_2_) ever synthesized are reported as weakly radioactive surrogates of therapeutic α‐emitters ^227^Th and ^230^U (Figure S1) [27, 28].

**FIGURE 1:**
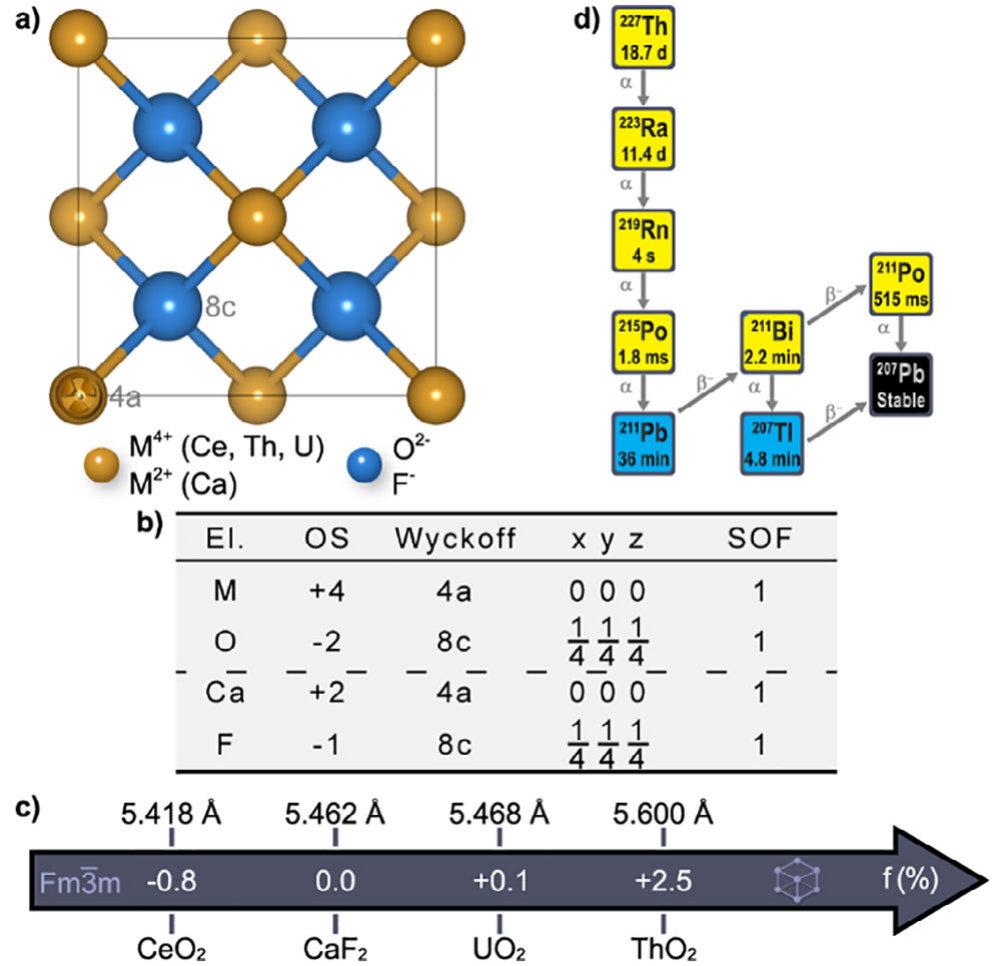
Crystal structure (a) and structural information (b) of isostructural cubic (space group #225 ‐ $\mathrm{Fm}\bar{3}m$) MO_2_ (M = Ce, Th, U) and CaF_2_ with their respective lattice parameters and lattice mismatch relative to CaF_2_ (c). Radioactive decay cascade of the therapeutic α‐emitter ^227^Th (clinical research only) (d).

CeO_2_ core NCs were first synthesized according to a modified thermal decomposition method as described in the Supporting Information (SI) [29]. The low magnification high‐angle annular dark‐field scanning transmission electron microscopy (HAADF‐STEM) image (Figure 2a) shows the formation of CeO_2_ NCs with some degree of size and shape polydispersity (7.4 ± 3.4 nm – 2σ standard deviation). Powder x‐ray diffraction (PXRD) confirmed the formation of NCs with a pure cubic crystal structure ($Fm\bar{3}m$) (Figure S2) with a calculated lattice parameter of 5.4144 (5) Å, which is nearly identical to its bulk counterpart (only 0.07% lattice contraction). High‐resolution transmission electron microscopy (HRTEM) confirmed the single crystalline nature of CeO_2_ NCs (Figures 2b and S3) while energy dispersive x‐ray (EDX) spectroscopy was used to confirm the co‐localization of Ce and O within NCs’ geometrical boundaries (Figure S4). As CeO_2_ can be easily reduced, especially in the nanoscale regime [30], the oxidation state of Ce was probed with Ce L_3_‐edge high‐resolution x‐ray absorption near‐edge spectroscopy (HR‐XANES) showing the predominance of Ce(IV) in the synthesized core NCs (Figure 2c) [31, 32], which is in perfect agreement with the PXRD data (Figure S2). Note that the quality of the starting CeO_2_ core NCs can be significantly improved by implementing a two‐phase solvothermal synthesis method, which is described in the SI. Nevertheless, the synthesis of highly monodisperse quasi‐spherical CeO_2_ core NCs (4.7 ± 1.4 nm – 2σ standard deviation, Figure S5) requests a much longer synthesis time (48 h instead of 1 h), which might reveal as a disadvantage when doping with therapeutic α‐emitters whose half‐life is just a few days.

**FIGURE 2:**
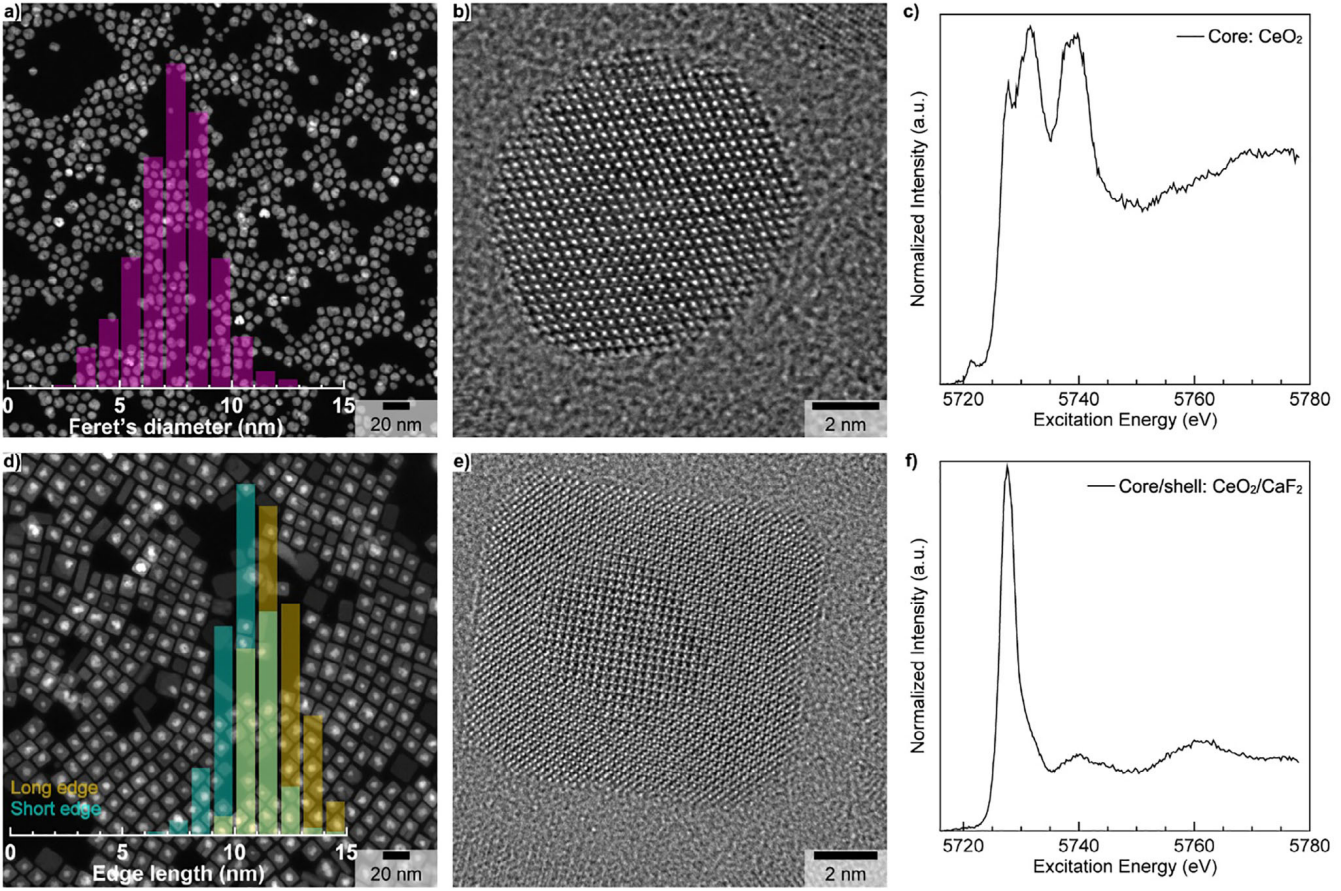
Low magnification HAADF‐STEM images (a, d) together with their corresponding overlaid size distribution histograms, HRTEM images (b, e**),** and Ce L_3_‐edge HR‐XANES spectra (c, f) of CeO_2_ core (a‐c) and CeO_2_/CaF_2_ core‐shell (d‐f) NCs. Size distribution histograms were obtained by measuring 1524 core (a) and 362 (d) core‐shell individual NCs.

Then, the obtained CeO_2_ core NCs were used as starting seeds for growing CeO_2 _/ CaF_2_ core‐shell NCs according to a modified protocol developed for the synthesis of short‐wavelength infrared emitting heterogeneous α‐NaYF_4_:Yb:Er:Ce / CaF_2_ core‐shell NCs for biological imaging [33]. The corresponding low magnification HAADF‐STEM image confirms the formation of nearly perfect cubic‐shape NCs (Figure 2d) with sizes for the short and long edges of 10.8 ± 5.2 nm and 12.0 ± 2.4 nm (2σ standard deviation), respectively.

The contrast difference between the anticipated core and shell domains in the HAADF‐STEM image (Figure 2d) was used to estimate the approximate size of the core region alone, which was found to be slightly smaller (6.7 ± 3.4 nm – 2σ standard deviation) compared to the initial size of the starting seeds (Figure S6). The PXRD pattern of the as‐prepared CeO_2 _/ CaF_2_ NCs (Figure S7) confirms that the cubic crystal structure is maintained after shell growth. The increase in the crystallite size is easily noticeable, and the lattice parameter expands by 1.3% compared to the starting CeO_2_ core NCs. The corresponding HRTEM image reveals perfect structural coherence, thus confirming the single‐crystalline nature of the synthesized heterostructure (Figure 2e).

Unexpectedly, the CaF_2_ shell deposition leads to almost complete reduction of Ce(IV) to Ce(III) (Figure 2f), which is not an effect of radiation beam damage (Figure S8). This behavior is reproducible and was observed for three different batches of CeO_2 _/ CaF_2_ NCs that were synthesized under different experimental conditions (Figure S9). Although the cubic symmetry is preserved after CaF_2_ shell deposition, the near‐complete reduction of Ce(IV) to Ce(III) is no longer compatible with the retention of the original chemical identity and/or chemical organization of the starting core NCs. The chemical and/or phase transition from CeO_2_ to either trigonal CeF_3_ ($P\bar{3}c1$) or trigonal Ce_2_O_3_ ($P\bar{3}m1$, type‐A) cannot explain the near‐complete reduction of Ce(IV) to Ce(III) because the experimental PXRD pattern (Figure S7) does not match with the theoretical ones (Figure S10a).

At this point, it is important to emphasize that the experimental PXRD pattern after shell deposition is a complex convolution of at least two PXRD patterns with (i) significant overlapping of Bragg peaks due to the size of the coherent domains, and (ii) structural coherence extending over chemically distinct domains. Although various cubic phases such as CeO_2_ ($Fm\bar{3}m$), CaF_2_ ($Fm\bar{3}m$), CeOF ($Fm\bar{3}m$), and type‐C Ce_2_O_3_ ($Ia\bar{3}$) are relatively good candidates, none of them can explain the experimental PXRD pattern (Figure S10b). As it is known that material combinations do not necessarily lead to the formation of core‐shell architectures [34, 35, 36, 37], meticulous chemical analyses were performed at the single particle level not only to confirm the formation of a core‐shell structure but also to explain the reduction of Ce(IV) to Ce(III). For simplicity, the synthesized heterostructure will be referred to as “CeO_2 _/ CaF_2_” in the rest of the manuscript.

First, a representative high‐resolution HAADF‐STEM image of an individual “CeO_2 _/ CaF_2_” NC clearly shows a sharp contrast difference between the innermost (brightest‐higher atomic number of Ce atoms) and outermost (darkest‐lower atomic number of Ca atoms) regions, indicating chemically distinct core and shell domains within a single NC (Figure 3a). Second, EDX elemental maps from an individual “CeO_2 _/ CaF_2_” NC were recorded, and an EDX line going through the center of the NC (Figure 3b) was selected and analyzed by implementing the subshell approach with spherical and cubic geometries for the core and shell regions, respectively [33, 34, 37, 38]. The raw (i.e., as determined after the quantification of the measured EDX spectra along the selected line scan) chemical profiles confirm the strong localization and high concentration of both Ce and O in the core region of the NC (Figure 3c). Applying the subshell approach to the raw normalized concentration profiles (Figure 3c) of the relevant cations and anions after data reduction reveals the formation of a core‐shell architecture with nearly pure oxide and fluoride domains that are separated by a 2‐3 nm highly intermixed interface (Figure 3d). The results are in very good agreement with those obtained for direct line scan measurements (Figure S11). The formation of Ca‐ and F‐rich shell as well as Ce‐ and O‐rich core domains is obvious. It is worth noting that whereas the core domain is pure (only Ce and O), the shell domain contains a tiny fraction of Ce (ca. 1 at.%) and O (ca. 2 at.%). The results obtained by EDX line scan analysis are in relatively good agreement with those obtained by the analysis of the FT pattern of the shell domain (Figure S12). Within the 2‐3 nm interface, the co‐existence of all cations and anions with chemical concentration gradients is a sign of the formation of a solid solution with numerous possibilities regarding chemical substitutions on Wyckoff positions 4a (cations) and 8c (anions). Nevertheless, it is currently impossible to distinguish between the formation of a homogeneously distributed solid solution with concentration gradients at the interface and the formation of structurally coherent but chemically distinct interphases that can be homogeneously or heterogeneously distributed at the interface.

**FIGURE 3:**
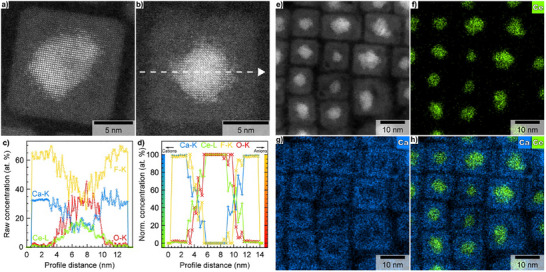
Raw (cropped only) high‐resolution HAADF‐STEM images of individual CeO_2_/CaF_2_ core‐shell NCs showing contrast difference between the anticipated core and shell domains (a, b). No image processing, altering/modifying the contrast of the HAADF‐STEM images was performed. Normalized raw (c) and calculated (i.e., implementing the subshell approach to raw elemental concentrations) chemical profiles obtained after the quantification of the EDX line‐scan (white arrow in b) for all cations and anions (d). HAADF‐STEM image (e) and corresponding EDX elemental maps of Ce (L line—**f**) and Ca (K line ‐ **g**) together with their overlap (h) for CeO_2_/CaF_2_ core‐shell NCs.

Finally, since line scan analysis probes only a limited region of an individual NC, EDX elemental mapping on the entire NCs region (Figure 3e) was employed to obtain the global distribution of Ce (Figure 3f) and Ca (Figure 3g). The overlap of the Ce and Ca chemical maps clearly proves the existence of the core‐shell architecture (Figure 3h). The detailed examination of the Ce chemical map confirms the presence of scattered Ce beyond the geometrical boundaries of the core and interface regions. This is clearly visible when overlapping the HAADF‐STEM image with the Ce chemical map (Figure S13). The quantification reveals that approximately 1‐2 at.% of Ce (normalized to the total quantity of metals, namely Ce and Ca) is distributed in the CaF_2_ shell, which is in good agreement with the results obtained from EDX line scan and HRTEM analyses. Although less defined compared to their cation counterparts, EDX elemental maps of O (Figure S14a) and F (Figure S14b) reveal that the former is preferentially located in the core regions (Figures S14c,e) while the latter is mostly located in the shell regions (Figures S14d,f), thus confirming the trend revealed by EDX line scan analysis. The structural and spectroscopic data combined with local chemical analyses indicate that the near‐complete reduction of Ce(IV) to Ce(III) is very likely driven by a topotactic vacancy‐driven reduction reaction leading to the transformation of the initial CeO_2_ core domain into a Ce^3+^‐rich oxygen‐deficient fluorite (i.e., CeO_2‐x_) and/or sesquioxide‐like phase (type‐C Ce_2_O_3_) with very limited influence on the integrity of the cation networks (see SI for Supporting Discussion). Although the experimental data presented constitute a solid body of evidence, this mechanistic interpretation, to be validated, will require additional structural characterization, which will be reported in a follow‐up study.

High‐quality “CeO_2_/CaF_2_” core‐shell NCs can also be synthesized when using ultra‐small CeO_2_ core NCs (Figure S15), and the CaF_2_ shell thickness can be tuned either by modifying the quantity of oleic acid (Figure S16) or the quantity of calcium precursor (Figure S17). The influence on the oxidation state of Ce and the atomic‐scale organization will have to be investigated. As cation intermixing is very limited (<5 at%) and doping of the reported core‐shell NCs with therapeutic α‐emitters will be in the range of 0.01 at.%, it is hypothesized that the effect on the retention of the radioactive decay products will be non‐existent or very limited (assuming a good stability of the host matrix under self‐irradiation). Nevertheless, that particular point will be of major interest and will have to be investigated in detail to determine an optimized and safe concentration of therapeutic α‐emitters per particle. Note that the as‐synthesized “CeO_2_/CaF_2_” NCs are only dispersible in non‐polar solvents but can be easily transferred into aqueous media by using PEG phospholipids (Figure S18) [33], thus paving the way towards bioconjugation for TAT application.

To demonstrate the relevance of the concept for TAT, we extended the synthesis strategy to additional materials systems, specifically AnO_2_ (An = U, Th) core NCs. These materials can be used as weakly radioactive surrogates to optimize the design and synthesis conditions prior to working with scarce and expensive therapeutic α‐emitters such as ^227^Th and ^230^U. Highly monodisperse (6.6 ± 0.8 nm – 2σ standard deviation) isotropic UO_2_ core NCs (depleted U) with a cubic crystal structure ($Fm\bar{3}m$) and a calculated lattice parameter of 5.4348 (6) Å were synthesized (Figure S19) and used as starting seeds for the epitaxial growth of CaF_2_ shell. The formation of cubic (Figure S20) nanocubes (short edge: 9.3 ± 1.6 nm, long edge: 10.6 ± 1.5 ‐ 2σ standard deviation) with segregated U‐rich core and Ca‐rich shell domains is revealed by HAADF‐STEM (Figure 4a) and EDX elemental mapping (Figure 4b–e). Although the formation of the core‐shell architecture is not questionable, it is obvious (Figure 4c,e) that U is intermixed into the CaF_2_ shell. This is confirmed by a 22% size reduction of the core domain with an average size of 5.1 ± 1.2 nm (2σ standard deviation) after shell growth (Figure S21) and a U content in the CaF_2_ shell of ca. 3 at.%. UO_2_, similarly to CeO_2_, can easily form non‐stoichiometric oxides and, in particular, hyper‐stoichiometric ones (UO_2+x_). Therefore, the near‐edge region (U M_4_‐edge) was used to probe the oxidation state of U in the synthesized UO_2_ core and UO_2 _/ CaF_2_ core‐shell NCs (Figure 4f). Compared to bulk UO_2_, the U M_4_‐edge spectrum of the core NCs is characterized by much greater intensity in the region of U(V/VI) (ca. 3726.5 eV). Similar oxidation behavior in nanoscale UO_2_ has been observed previously [40, 41]. However, after CaF_2_ shell growth, the U M_4_‐edge spectral profile is more similar to that of bulk UO_2_, implying the stabilization of U(IV) by the CaF_2_ shell, which is not due to radiation beam damage (Figure S22). Additional weaker features at 3728.8 and 3732.6 eV are analyzed in the SI (Figure S23). The synthesis method is also adapted when replacing UO_2_ by ultra‐small (3.7 ± 1.0 nm – 2σ standard deviation) isotropic ThO_2_ (natural Th) core NCs (Figures 5a and S24). Despite the ultra‐small size of the starting ThO_2_ seeds, highly homogeneous cubic‐shape ThO_2 _/ CaF_2_ NCs (short edge: 13.2 ± 2.2 nm, long edge: 15.6 ± 2.8 nm, and 12.0 ± 2.4 nm – 2σ standard deviation) are formed (Figures 5b and S25, S26). Although the core‐shell structure is confirmed by the sharp contrast difference between the Th‐rich (core) and Ca‐rich (shell) domains on the HAADF‐STEM image, up to 2 at.% of Th was intermixed into the CaF_2_ shell. Indeed, a 19% size reduction of the core domain down to 3.0 ± 1.6 nm (2σ standard deviation) is observed after shell growth (Figure 5b). It is worth noting that the size reduction of the core domain after shell growth is very similar to the one observed for UO_2_, despite the initial ThO_2_ core NCs being less than half the size of the UO_2_ starting seeds.

**FIGURE 4:**
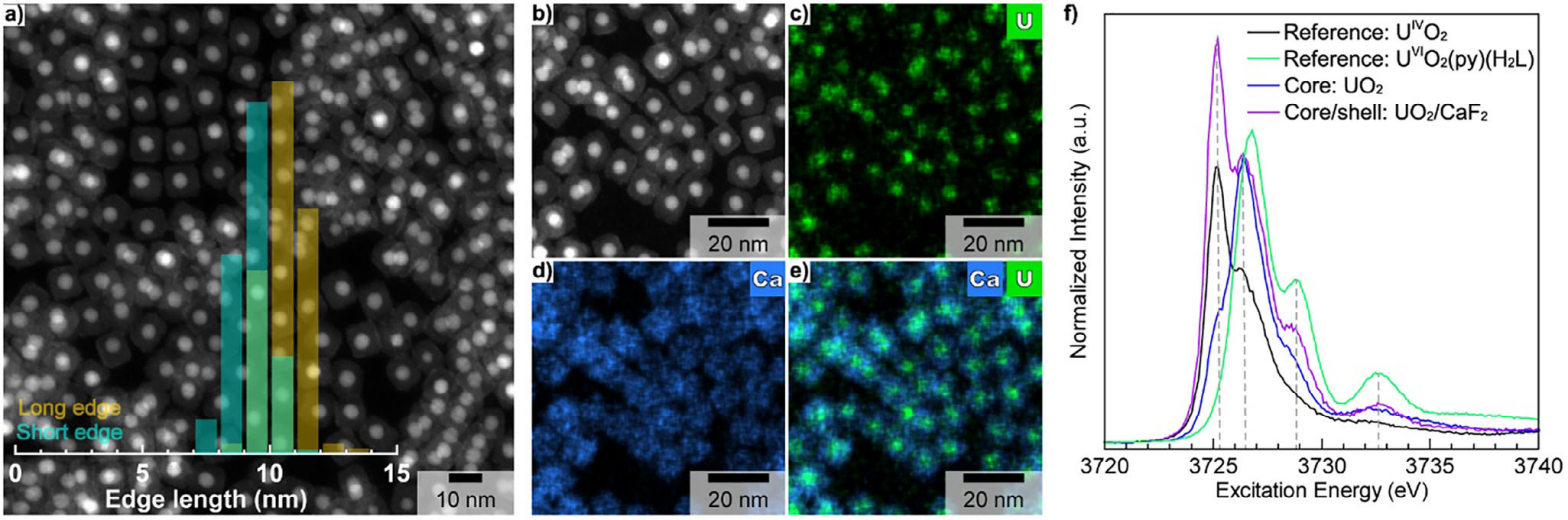
Low magnification HAADF‐STEM image together with its corresponding overlaid size distribution histograms of UO_2_/CaF_2_ core‐shell NCs (a). Size distribution histograms were obtained on 162 individual core‐shell NCs. HAADF‐STEM image (b) and corresponding EDX elemental maps of U (M line—**c**), Ca (K line ‐ **d**) together with their overlap (e) for UO_2_/CaF_2_ core‐shell NCs. U M4‐edge HR‐XANES spectra of bulk UO_2_ (black solid line), UO_2_(py)(H_2_L) (L = polypyrrolic Schiff‐base) [39] (green solid line), UO_2_ core (blue solid line), and UO_2_/CaF_2_ core‐shell (violet solid line) NCs (f).

**FIGURE 5:**
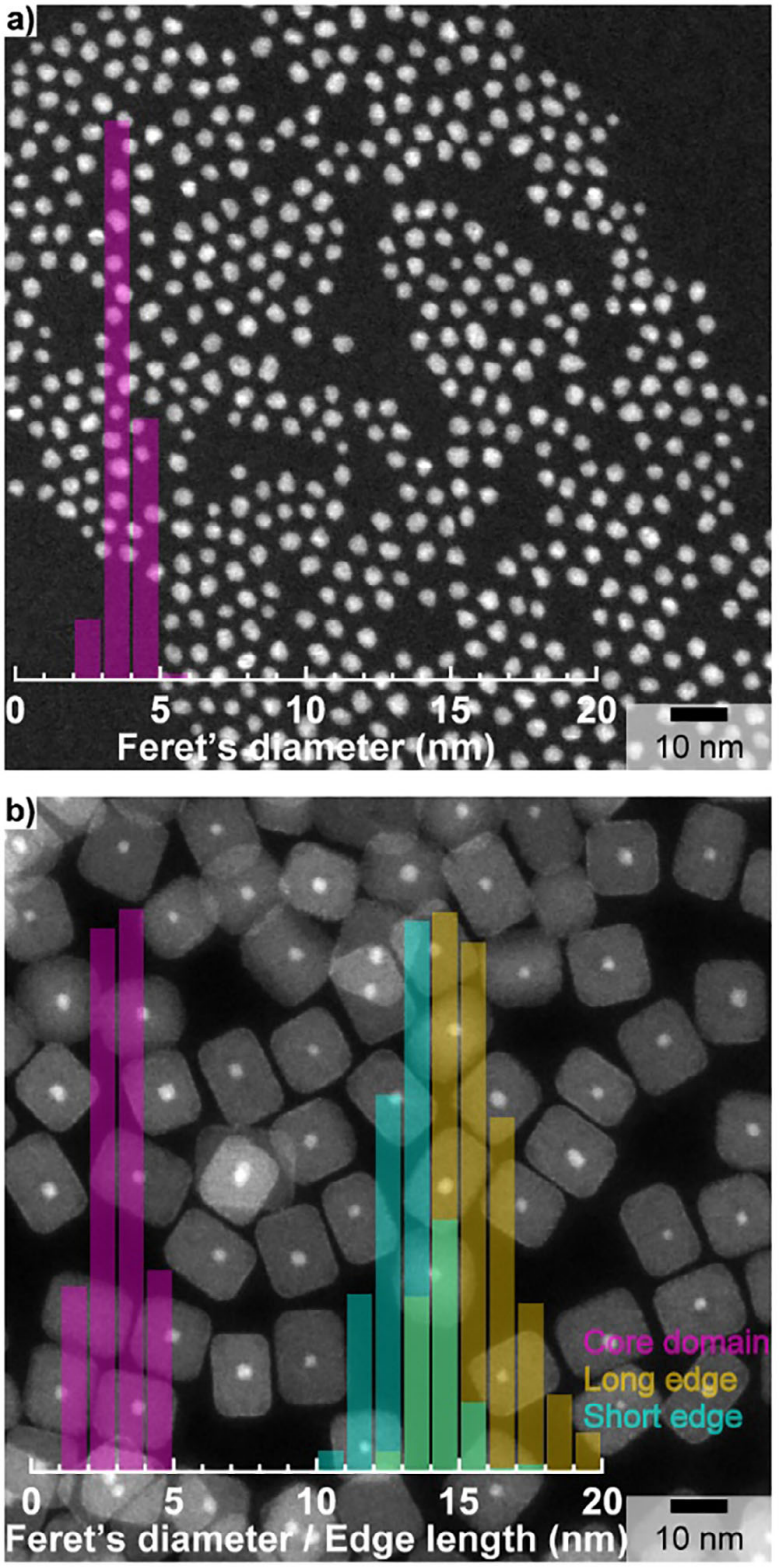
Low magnification HAADF‐STEM images together with their corresponding overlaid size distribution histograms (a, b), of ThO_2_ core (a) and ThO_2_/CaF_2_ core‐shell (b) NCs. Size distribution histograms were obtained by measuring 1067 core (a) and 409 (b) core‐shell individual NCs.

In conclusion, we have synthesized and characterized a novel class of high‐quality heterostructured CeO_2‐x _/ CaF_2_ (or Ce_2_O_3_/CaF_2_) and AnO_2_/CaF_2_ (An = U, Th) core‐shell NCs that have never been reported in the literature. Although the formation of these heterostructures is not questionable, the growth mechanism is more complex than simple epitaxial growth as initially anticipated. Beyond their synthetic novelty or fundamental relevance, and although no therapeutic evaluation is reported in this communication, these novel NCs not only constitute promising lead candidates but also well‐defined model systems for the systematic investigation of essential preclinical “stage‐gates” that currently prevent the emergence of core‐shell NCs as an additional option in the TAT toolbox. In our opinion, critical “stage‐gates” include (i) the incorporation of clinically relevant α‐emitters (primarily ^227^Th/^223^Ra, ^230^U but also ^225^Ac) in the core‐shell heterostructures, (ii) the optimization of radiolabeling yields, and (iii) the response of the core–shell structures to self‐irradiation and their ability to efficiently confine radioactive decay products as a function of the atomic‐scale organization/chemical composition and shell thickness. These critical investigations must ultimately be evaluated alongside comprehensive toxicity assessments. This work lays the groundwork for rationally engineered inorganic core–shell NCs to define the therapeutic window and realistic clinical potential of such materials for locoregional TAT.

## Conflicts of Interest

The authors declare no conflicts of interest.

## Supporting information




**Supporting File 1**: anie71458‐sup‐0001‐SuppMat.docx.

## Data Availability

The data that support the finding of this study are available in the Supporting Information of this article. Raw data are available, upon reasonable request, by addressing an e‐mail to Damien Hudry.
