# Supplementary material for: Controlled Synthesis of a New Class of Heterostructured Metal Oxides (Cerium, Thorium, Uranium)/Calcium Fluoride Core‐Shell Nanocrystals With Atomically Coherent Interfaces
Source: Angew Chem Int Ed Engl. 2026 Feb 15;65(12):e24282. doi: 10.1002/anie.202524282 (PMC12991035; doi:10.1002/anie.202524282)
Supplement: Supplementary file 1 — Supporting File 1: anie71458‐sup‐0001‐SuppMat.docx. [file ANIE-65-e24282-s001.docx]

SUPPORTING INFORMATION

**Controlled Synthesis of a New Class of Heterostructured Metal Oxides (Cerium, Thorium, Uranium) / Calcium Fluoride Core-Shell Nanocrystals with Atomically Coherent Interfaces**

*Dejing Meng,^1^ Radian Popescu,^2^ Carmen M. Andrei,^3^ Jérôme Himbert,^4^ Eduard Madirov,^5^Jacob A. Branson,^6^ Emily M. Reynolds,^6^ Tim Prüßmann,^6^ Jörg Göttlicher,^7^ Tonya Vitova,^6^ Niko Hildebrandt,^5^ Yolita Eggeler,^2^ Bryce S. Richards,^1, 8^ Olaf Walter,^4^ and Damien Hudry.^1^**

^1^ Institute of Microstructure Technology, Karlsruhe Institute of Technology, Karlsruhe – Germany

^2^ Laboratory for Electron Microscopy, Karlsruhe Institute of Technology, Karlsruhe – Germany

^3^ Canadian Center for Electron Microscopy, McMaster University, Hamilton, ON, L8S 4M1 – Canada

^4^ European Commission, Joint Research Centre (JRC), Karlsruhe, Germany

^5^ Department of Engineering Physics, McMaster University, Hamilton, ON, M8S 4K1 – Canada

^6^ Institute for Nuclear Waste Disposal, Karlsruhe Institute of Technology, Karlsruhe – Germany

^7^ Institute for Photon Science and Synchrotron Radiation, Karlsruhe Institute of Technology, Karlsruhe – Germany

^8^ Light Technology Institute, Karlsruhe Institute of Technology, Karlsruhe – Germany

* Corresponding author

**1. Materials**

Ammonium cerium (IV) nitrate ((NH_4_)_2_Ce(NO_3_)_6_, ≥98%), cerium(III) nitrate hexahydrate (Ce(NO_3_)_3_•6H_2_O, 99%), trifluoroacetic acid (CF_3_COOH – TFAH, 99%), oleylamine (OAm, ≥ 98% primary amine), were purchased from Merck. Octadecene (ODE, technical grade 90%) was purchased from Thermo Scientific. Absolute ethanol (EtOH, laboratory reagent grade ≥99%), toluene (C_6_H_5_CH_3_, analytical reagent grade ≥99.8%), acetone (CH_3_OCH_3_, analytical reagent grade ≥99.8%) and oleic acid (OA, purified ≥ 79%) were purchased from VWR Chemicals. Calcium carbonate (CaCO_3_, 99.99%), was purchased from Alfa Aesar. 18:1 PEG2000 PE (Avanti Polar Lipids) was purchased from Merck. Uranyl acetylacetonate (UO_2_(acac)_2_ – depleted uranium) was purchased from International Bio-Analytical Industries Inc. (Boca Raton, Florida, USA). Thorium nitrate ([Th(NO_3_)_4_•5H_2_O]) was purchased from Merck. All chemicals were used as received without further purification. (NH_4_)_2_Ce(NO_3_)_6_, ODE, OA, OAm are stored under inert conditions inside a glovebox under nitrogen (O_2_ and H_2_O levels < 1 ppm).

**2. Synthesis details**

**Synthesis of calcium trifluoroacetate (Ca(OOCCF_3_)_2_)**

Ca(OOCCF_3_)_2_ was prepared by adding 10 mmol (10.01 g) of CaCO_3_ together with 45 mL of deionized water (DI) in a 100 mL round bottom flask equipped with a reflux condenser. Then, dropwise addition of 22 mL (287.5 mmol) of TFAH was performed at room temperature under stirring. Instantaneous release of CO_2_ was observed and a perfectly clear colorless solution was obtained. The latter was heated up to 90°C under air and kept overnight under continuous stirring. After cooling, the solution was filtered (602 H½ filter paper) and the solvent evaporated with a rotary evaporator at 70°C under reduced pressure (80 mbar) for 180 min. The obtained wet powder was dried under vacuum at 150°C for 24 hours. Immediately after drying, the powder was transferred into a glovebox under dry nitrogen.

**Synthesis of CeO_2_ core nanocrystals: thermal decomposition method**

In a glovebox under dry nitrogen, 0.5 mmol of (NH_4_)_2_Ce(NO_3_)_6_ (274.3 mg) are introduced in a 50 mL three-neck round bottom Schlenk flask together with 28.3 mmol of ODE (9 mL), and 3 mmol of OAm (1 mL). The resulting mixture is transferred to a Schlenk line and purged at room temperature by performing five argon (Ar) ↔ vacuum (5.10^-2^ mbar) cycles. Then, the slurry is heated up to 120°C (under Ar flow) to dissolve (NH_4_)_2_Ce(NO_3_)_6_. The temperature is maintained for 30 min during which the turbid solution turns to an optically clear orange solution. The obtained solution is purified under vacuum at 120°C by performing five Ar ↔ vacuum (5.10^-2^ mbar) cycles. Finally, the solution is heated up under Ar to 200°C and maintained for 15 min. When the temperature reaches 200°C, the solution immediately turns dark-brown. Then, the heating mantle is removed, and the flask is cooled down with a flow of compressed air. After cooling, the NCs are extracted and purified by implementing the procedure that includes i) acetone precipitation, ii) centrifuging (6797xg), iii) discarding of the supernatant, and iv) precipitate redispersion in 1 mL toluene. The procedure is repeated 3 times. The resulting dark-brown solution is stored in a tightly closed glass vial and utilized as a stock solution for the subsequent CaF_2_ shell deposition.

**Synthesis of CeO_2_ core nanocrystals: solvothermal method**

In a glovebox under dry nitrogen, Ce(NO_3_)_3_•6H_2_O (0.125 mmol – 54.3 mg) is introduced in a 10 mL glass vial with a screw cap. The glass vial is removed from the glovebox and the Ce(III) nitrate powder is dissolved in 7.5 mL DI water. This constitutes the aqueous phase. For the organic phase, 7125 μL of toluene are introduced in a 10 mL glass vial (equipped with a screw cap) together with 0.942 mmol OA (300 μL) and 0.7 mmol of tert-butylamine (75 μL). The resulting solution is vortexed for 10 seconds. Then, the clear colorless aqueous phase is carefully transferred in a 23 mL PTFE liner with a 10 mL disposable syringe connected to a 120 mm disposable needle (gauge: 21). During this step, it is important to avoid the formation of water droplets on the PTFE liner wall especially on its top part. Afterwards, another 10 mL disposable syringe connected to a 120 mm disposable needle (gauge: 21) is used to add the organic solution on top of the aqueous phase in the PTFE liner. It is important to avoid the formation of an emulsion. When the organic phase is successfully transferred, a perfectly clear colorless 2-phase system is visible in the PTFE liner. The latter is carefully introduced in a 23 mL stainless steel autoclave (Parr Instrument) that is sealed according to manufacturer’s guidelines. The sealed autoclave is introduced in a heating block at room temperature. Finally, the autoclave is heated up to 180°C and kept at this temperature for 48 hours. After 48 hours, the autoclave is removed from the heating block and cooled down to room temperature under a strong flow of compressed air. After cooling, the organic phase (brownish) is carefully removed and introduced in a 50 mL centrifuge tube. The CeO_2_ NCs are extracted and purified with the same procedure as described for the thermal decomposition method.

**Synthesis of CeO_2_/CaF_2_ core-shell nanocrystals**

In a glovebox under dry nitrogen, 1 mmol of Ca(OOCCF_3_)_2_ (266.1 mg) are introduced in a 50 mL three-neck round bottom Schlenk flask together with 32.8 mmol of ODE (10.5 mL), and 11 mmol of OA (3.5 mL). The resulting mixture is transferred to a Schlenk line and 200 μL of the stock solution of CeO_2_ core NCs (91.8 mg/mL – including both the organic and inorganic parts) are added. The obtained slurry is purged at room temperature by performing five Ar ↔ vacuum (5.10^-2^ mbar) cycles. Then, the slurry is heated up to 120°C (under Ar flow) to dissolve Ca(OOCCF_3_)_2_. The temperature is maintained for 30 min during which the turbid solution turns to an optically clear gold-like solution, which is purified under vacuum at 120°C by performing five Ar ↔ vacuum (5.10^-2^ mbar) cycles. Finally, the solution is heated up under Ar to 300°C and maintained for 45 min. Then, the heating mantle is removed, and the flask is cooled down with a flow of compressed air. After cooling, the NCs are extracted and purified by implementing the procedure that includes i) acetone precipitation, ii) centrifuging (6797xg), iii) discarding of the supernatant, and iv) precipitate redispersion in 1 mL toluene. The procedure is repeated 3 times. The resulting slightly yellowish/brownish solution is stored in a tightly closed glass vial.

**Synthesis of CeO_2_/CaF_2_ core-shell nanocrystals with modified shell thickness**

The exact same protocol as described in the previous section is implemented but either the quantity of OA (Method 1) or the quantity of calcium trifluoroacetate (Method 2) are modified. All quantities are given in Table S1.

| Method 1 | | |
| --- | --- | --- |
| Ca(OOCCF_3_)_2_ | ODE | OA |
| 1 mmol | 12.5 mL | 1.5 mL |
| 1 mmol | 7 mL | 7 mL |
| Method 2 | | |
| 0.5 mmol | 12.25 mL | 1.75 mL |
| 2 mmol | 7 mL | 7 mL |

***Table S1.*** *Molar and volumetric quantities used to modify the CaF_2_ shell thickness.*

**Water transfer of CeO_2_/CaF_2_ core-shell nanocrystals**

11.35 mg of core-shell NCs are introduced in a 4 mL glass vial. Note that the given mass is the total mass including both the NCs and the stabilizing organic ligands (oleates). Then, 568 μL of a 200 mg/mL solution of 18:1 PEG2000 PE phospholipids dispersed in chloroform are added. The NCs are easily redispersed (within less than 5 minutes at 30°C) and a perfectly clear slightly brownish solution is obtained. Chloroform is then evaporated under a gentle Ar flow at ca. 50°C. Chloroform evaporation takes approximately 70 minutes and a white/translucid solid is obtain after cooling down to RT. Note that when reheating (*ca.* 50°C), a clear but highly viscous and slightly brownish liquid is obtained. After melting, DI water (1336 μL) is slowly added. The obtained solution is sonicated for 30 minutes at 30°C. The obtained colloidal suspension is stored at room temperature and stable for at least 10 days (maximum time available to monitor the stability before submitting the revised version of the manuscript).

**Synthesis of UO_2_ core nanocrystals**

**Caution!** Although depleted uranium was used with a relatively low activity, usual precautions for working with radioactive elements must be followed. All synthesis experiments involving depleted uranium were performed in a dedicated nuclear facility at the Joint Research Center (JRC) Karlsruhe of the European Commission.

UO_2_ core NCs were synthesized by modifying the procedure initially reported by Hudry and co-workers.^[1]^ 0.4 mmol of UO_2_(acac)_2_ (187.5 mg) are introduced in a 50 mL three-neck round bottom Schlenk flask together with 42 mmol of BnOBn (8 mL), 4.1 mmol of OA (1.3 mL), and 4.1 mmol of OAm (1.35 mL). The resulting mixture is purged at room temperature by performing five argon (Ar) ↔ vacuum (5.10^-2^ mbar) cycles. Then, the slurry is heated up to 120°C (under Ar flow) and the temperature is maintained for 20 min. The obtained solution is purified under vacuum at 120°C by performing five Ar ↔ vacuum (5.10^-2^ mbar) cycles. The resulting solution is first heated up under Ar to 220°C and maintained for 10 min during which an optically orange-brown solution is obtained, which then turns black. The black solution is then heated up under Ar to 280°C and maintained for 20 min. Then, the heating mantle is removed, and the flask is cooled down with a flow of compressed air. After cooling, the NCs are extracted and purified by implementing the procedure that includes i) acetone/ethanol precipitation, ii) centrifuging (6797xg), iii) discarding of the supernatant, and iv) precipitate redispersion in 2 mL toluene. The procedure is repeated 3 times. The resulting dark-brown solution is stored in a tightly closed glass vial and utilized as a stock solution for the subsequent CaF_2_ shell deposition.

**Synthesis of UO_2_/CaF_2_ core-shell nanocrystals**

**Caution!** Although depleted uranium was used with a relatively low activity, usual precautions for working with radioactive elements must be followed. All synthesis experiments involving depleted uranium were performed in a dedicated nuclear facility at the Joint Research Center (JRC) Karlsruhe of the European Commission.

In a glovebox under dry nitrogen, 1 mmol of Ca(OOCCF_3_)_2_ (266.1 mg) are introduced in a 50 mL three-neck round bottom Schlenk flask together with 32.8 mmol of ODE (10.5 mL), and 10 mmol of OA (3.2 mL). The resulting mixture is transferred to a Schlenk line and 1000 μL of the stock solution of UO_2_ core NCs (35 mg/mL – including both the organic and inorganic parts) are added. The obtained slurry is purged at room temperature by performing five Ar ↔ vacuum (5.10^-2^ mbar) cycles. Then, the slurry is heated up to 120°C (under Ar flow) to dissolve Ca(OOCCF_3_)_2_. The temperature is maintained for 30 min and the resulting black solution is purified under vacuum at 120°C by performing five Ar ↔ vacuum (5.10^-2^ mbar) cycles. Finally, the solution is heated up under Ar to 300°C and maintained for 60 min. Then, the heating mantle is removed, and the flask is cooled down with a flow of compressed air. After cooling, the NCs are extracted and purified by implementing the procedure that includes i) acetone/ethanol precipitation, ii) centrifuging (6797xg), iii) discarding of the supernatant, and iv) precipitate redispersion in 1 mL toluene. The procedure is repeated 3 times. The resulting clear black solution is stored in a tightly closed glass vial.

**Synthesis of ThO_2_ core nanocrystals**

**Caution!** Although natural thorium was used with a relatively low activity, usual precautions for working with radioactive elements must be followed. All synthesis experiments involving natural thorium were performed in a dedicated nuclear facility at the Joint Research Center (JRC) Karlsruhe of the European Commission.

As well-defined ThO_2_ core NCs cannot be synthesized by the classical thermal decomposition method,^[1, 2]^ a solvothermal method, initially developed for CeO_2_ NCs,^[3, 4]^ was adapted for the synthesis of ThO_2_ core NCs.

80 mg Th(NO_3_)_4_.5H_2_O are dissolved in 1mL of deionized water. The resulting aqueous solution is added to a 23 mL Teflon liner and an organic mixture (10 mL of tolunene, 2 mL of OA, 150 μl of Terbutylamine) is carefully added to get a 2-phase liquid system. The Teflon liner is then added in a stainless steel autoclave, which is sealed under air. The autoclave is heated at 200°C for 48h. After cooling, the organic phase is carefully removed from the Teflon liner and the NCs are extracted and purified by implementing the procedure that includes i) acetone/ethanol precipitation, ii) centrifuging (6797xg), iii) discarding of the supernatant, and iv) precipitate redispersion in 1 mL toluene. The procedure is repeated 3 times. The resulting clear colorless solution is stored in a tightly closed glass vial.

**Synthesis of ThO_2_/CaF_2_ core-shell nanocrystals**

**Caution!** Although natural thorium was used with a relatively low activity, usual precautions for working with radioactive elements must be followed. All synthesis experiments involving natural thorium were performed in a dedicated nuclear facility at the Joint Research Center (JRC) Karlsruhe of the European Commission.

The exact same protocol as for UO_2_/CaF_2_ core-shell NCs was used except that UO_2_ core NCs were replaced by ThO_2_ core NCs.

**3. Instruments and methods**

**Powder X-ray diffraction (PXRD)**

PXRD was performed at room temperature in Bragg-Brentano geometry using a Bruker D8 Discover powder diffractometer with a copper anticathode, a quartz monochromator (Cu Kα1 radiation, λ=1.540562 Å), and a 1-dimensional detector (LynxEye XE-T). PXRD patterns were recorded in the 2θ range 10°-135° for 9h. Pawley refinements were performed by using TOPAS software (version 7). PXRD samples were prepared by drop-casting colloidal suspensions of the core or core-shell NCs precipitated in acetone onto low background (911)-oriented silicon substrates.

**Electron Microscopy (Osiris): radioactive and non-radioactive nanocrystals**

High-angle annular dark field (HAADF) scanning transmission electron microscopy (STEM) combined with energy dispersive X-ray (EDX) spectroscopy experiments were carried out with a FEI Osiris ChemiSTEM microscope (operated at 200 keV electron energy), which is equipped with a Super-X EDX system (comprising four silicon drift detectors - Bruker XFlash) for EDX spectroscopy. EDX spectra are quantified with the FEI software package “TEM imaging and analysis” (TIA) version 4.7 SP3. Using TIA, element concentrations were calculated on the basis of a refined Kramers’ law model, which includes corrections for detector absorption and background subtraction. For this purpose, standard-less quantification (theoretical sensitivity factors) without thickness correction was applied. The quantification of F-, O-, Ce-, and Ca-content from their EDX spectra (line scans) was performed by evaluating the X-ray characteristic intensities of the F-K_α_ line, O-K_α_ line, Ce-L and Ca-K series. X-ray lines of Cu (K- and L-series) from the TEM grid as well as the C-K_α_ line from the amorphous carbon substrate were always present in the EDX spectra. Grids were prepared at room temperature in air by drop casting 10 μL of a diluted suspension of NCs in toluene onto an ultrathin amorphous carbon film (3 nm) on holey carbon support film mounted on 400 μm mesh Cu grid (Ted Pella Inc.).

Concentration profiles of different elements within a single NC were obtained from EDX spectra acquired along a line that passes through the center of the corresponding NC. EDX line profiles were recorded by applying a drift-correction routine via cross correlation of several images, which yields a local precision better than 1 nm. The drift-corrected EDX line profiles were acquired with a probe diameter of 0.4 nm and a distance of 1 nm between two measuring points along the line. The mathematical treatment applied to raw chemical concentration profiles was described in several of our publications, including for the sphere in a cube geometry.^[5-7]^

For radioactive samples, EDX elemental maps were recorded and used to determine the distribution of actinides within the core-shell structures. EDX elemental maps were analyzed by using the ESPRIT software from Bruker (version 2.3).

**Electron Microscopy (Spectra Ultra): non-radioactive nanocrystals**

Diluted NCs were spread on an ultrathin carbon film on lacey carbon support film, 400 mesh, copper grid (Ted Pella Inc.). To remove any contamination, the grids were baked overnight at 100°C.

The samples were analyzed with the Spectra Ultra (Thermo Fisher Scientific), double-corrected HRTEM/STEM operated at 300kV. EDX elemental maps were acquired using the Ultra X detector equipped with six Super-X silicon drift detectors (SDD) using a probe current of 100 pA. High Angle Annular Dark Field (HAADF) images were acquired in STEM mode with a probe current of 50 pA and 28 mrad convergence angle, and a probe size of 60 pm. The pixel size for the frames varied from 25 to 50 pm, and the dwell time was 5 μs. The chemical maps and EDX line scans were generated using Velox program from Thermo Fisher Scientific (version 3.16.1).

Quantified EDX elemental maps were further evaluated by selecting line scans going through the centre of single NCs, thus resulting in raw concentration profiles along the investigated particles. Then, the same mathematical treatment as described for line scans performed with the OSIRIS was performed (*i.e.* sub-shell approach) to the raw concentration profiles to determine elemental concentrations independently of the shell.

**High Resolution X-ray Absorption Near Edge Structure**

Non-radioactive samples: CeO_2_ and CeO_2_/CaF_2_ NCs were prepared by drop-casting the concentrated colloidal solution into a cryogenic-compatible holder with four sample positions and allowing the solution to evaporate. The holder was sealed with a 13 µm Kapton window.

The HR-XANES spectra at the Ce L_3_-edge were recorded at the SUL-X beamline at the KIT Light Source in Karlsruhe, Germany. The SUL-X beamline is operated with a 27-pole wiggler as the radiation source. The incoming beam was monochromatized with a Si(111) fixed-exit double-crystal monochromator, and the resulting beam size was about 0.2 mm horizontally by 1.2 mm vertically. The emission energy was selected using the [422] reflection of one spherically bent Si crystal analyzer using a 0.5 m bending radius. Signal was detected in fluorescence mode with a 7-element silicon drift detector (Sirius, RaySpec) using the Ce L_3_N_4,5_ fluorescence line. Measurements were performed under high vacuum (10^−5^ mbar) to minimize the absorption by air of the low energy X-ray radiation. Tests for radiation damage were performed by collecting a series of quick HR-XANES spectra and monitoring the spectral profile as a function of beam exposure time, with changes in the spectral profile indicating beam damage. Measurements were performed using a liquid nitrogen cryostat in order to minimize radiation damage. Subsequent measurements on undamaged sample regions were kept below the time needed to prevent damage. Energy calibration was performed using the first maximum of the first derivative of the vanadium K-edge HR-XANES spectrum of vanadium foil (5465.1 eV). For background subtraction, a line with the slope set to zero was fit to the pre-edge region from 5707.4 to 5715.0 eV, and this line was subtracted from the entire spectrum. Both the pre-edge and post-edge regions are approximately linear with slopes of zero. To normalize the spectra, a line was fit in the approximately-linear post-edge region from 5770.0 to 5777.6 eV with a slope set to zero. The spectra were divided by the y-intercept of the line, resulting in a post-edge intensity of one 1.0 for each spectrum.

Radioactive samples: UO_2_ and UO_2_/CaF_2_ NCs were prepared by drop-casting the concentrated colloidal solution into a cryogenic-compatible holder with three sample positions and allowing the solution to evaporate. A 13 µm Kapton window was used for primary containment. This holder was placed into a larger holder with another 13 µm Kapton window for secondary containment.

The reference of bulk UO_2_ was prepared as a pellet, doubly contained in 13 µm Kapton. Both NCs and the bulk UO_2_ reference were measured during the same experiment day and under the same experimental conditions.

The HR-XANES spectra at the U M_4_-edge were collected at the CAT-ACT beamline of the KIT Light Source in Karlsruhe, Germany. The incident energy was selected using the [111] reflection from a double Si crystal monochromator. The estimated flux at the sample position was on the order of 10^9^ photons/s at an incident energy of 3.8 keV. The size of the incident beam was defined to 500 µm vertically by placement of a slit in front of the sample. The use of horizontal slits does not improve the experimental resolution, so none were used, leading to a horizontal beam size of approximately 1000 µm. The HR-XANES spectra were obtained by recording the maximum intensity of the U M_4_N_6_ emission line (3337.0 eV) as a function of the incident energy. The emission energy was selected using the [220] reflection of one spherically bent Si crystal analyzer (Saint-Gobain) with a 1 m bending radius and 90° scattering geometry that has been aligned to the 75° Bragg angle. A Johann-type X-ray emission spectrometer with a 1 m diameter equal to the bending radius of the analyzer crystal in focusing geometry was used. The emitted fluorescence was focused onto and detected by a single diode VITUS H150 silicon drift detector (KETEK, Germany). The entire spectrometer environment was contained within a He-purged glovebox due to the high attenuation of tender X-rays by air. Measurements were performed at room temperature. Tests for radiation damage was performed by monitoring for changes in spectral profile as a function of incident beam exposure time. An energy range from 3712.0 to 3822.5 eV was scanned with a step size down to 0.1 eV with a dwell time of 5 s across the absorption edge. Energy calibration was performed by reference to the bulk UO_2_ pellet for which the maximum of the white line intensity is calibrated to 3725.2 eV. To normalize the spectra, a line was fit in the approximately-linear post-edge region from 3773.0 to 3822.5 eV with a slope set to zero. The spectra were divided by the y-intercept of the line, resulting in a post-edge intensity of one 1.0 for each spectrum.

**4. Supplementary figures**


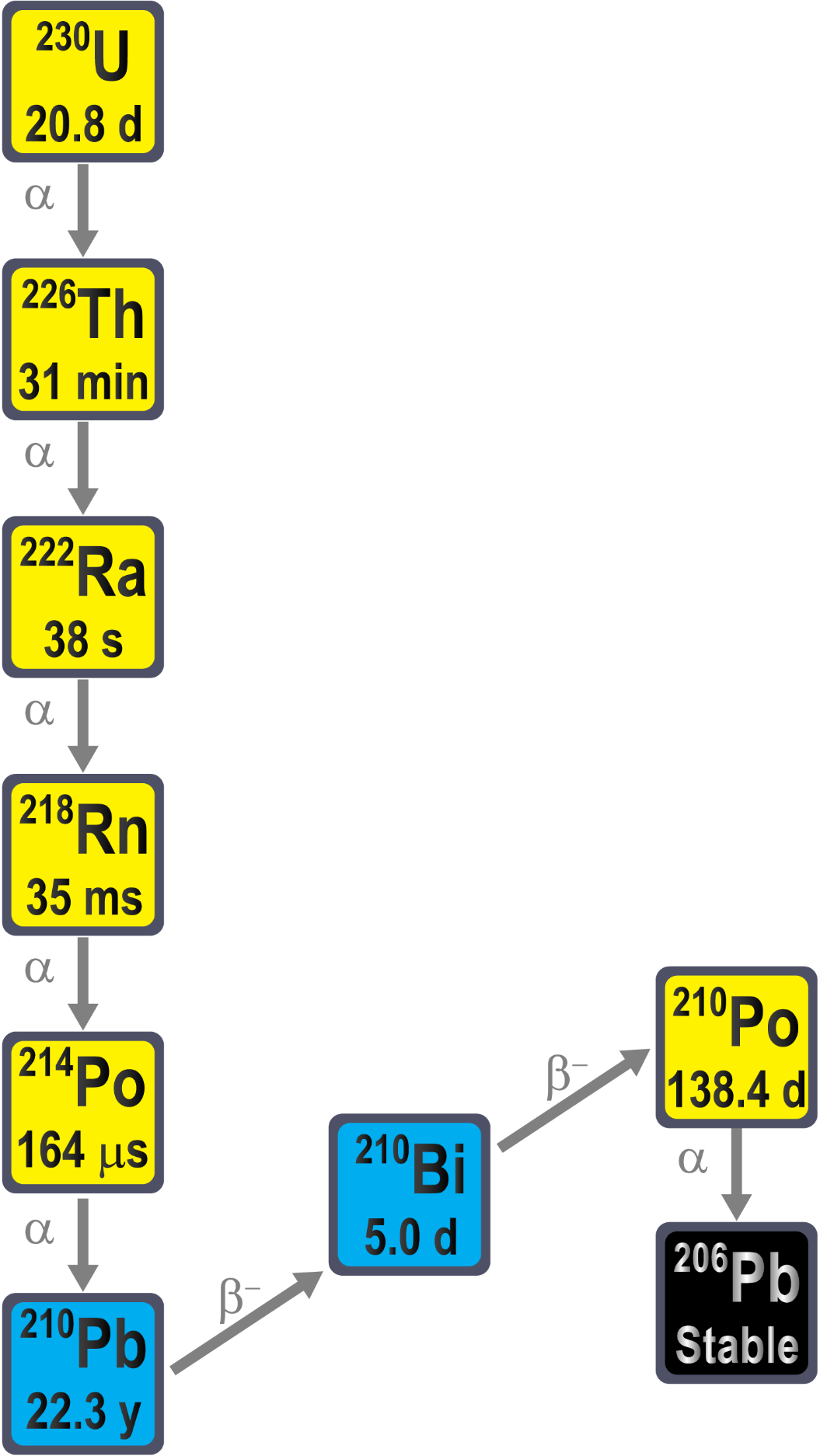


**Figure S1.** Radioactive decay cascade of the therapeutic α-emitter ^230^U (clinical research only). ^230^U is a pure α-emitter decaying through a cascade of four further α-emitters to long-lived ^210^Pb. The decay of ^230^U generates five α-particles with a cumulative energy of 33.5 MeV, delivering a highly cytotoxic dose to targeted cells.^[8]^


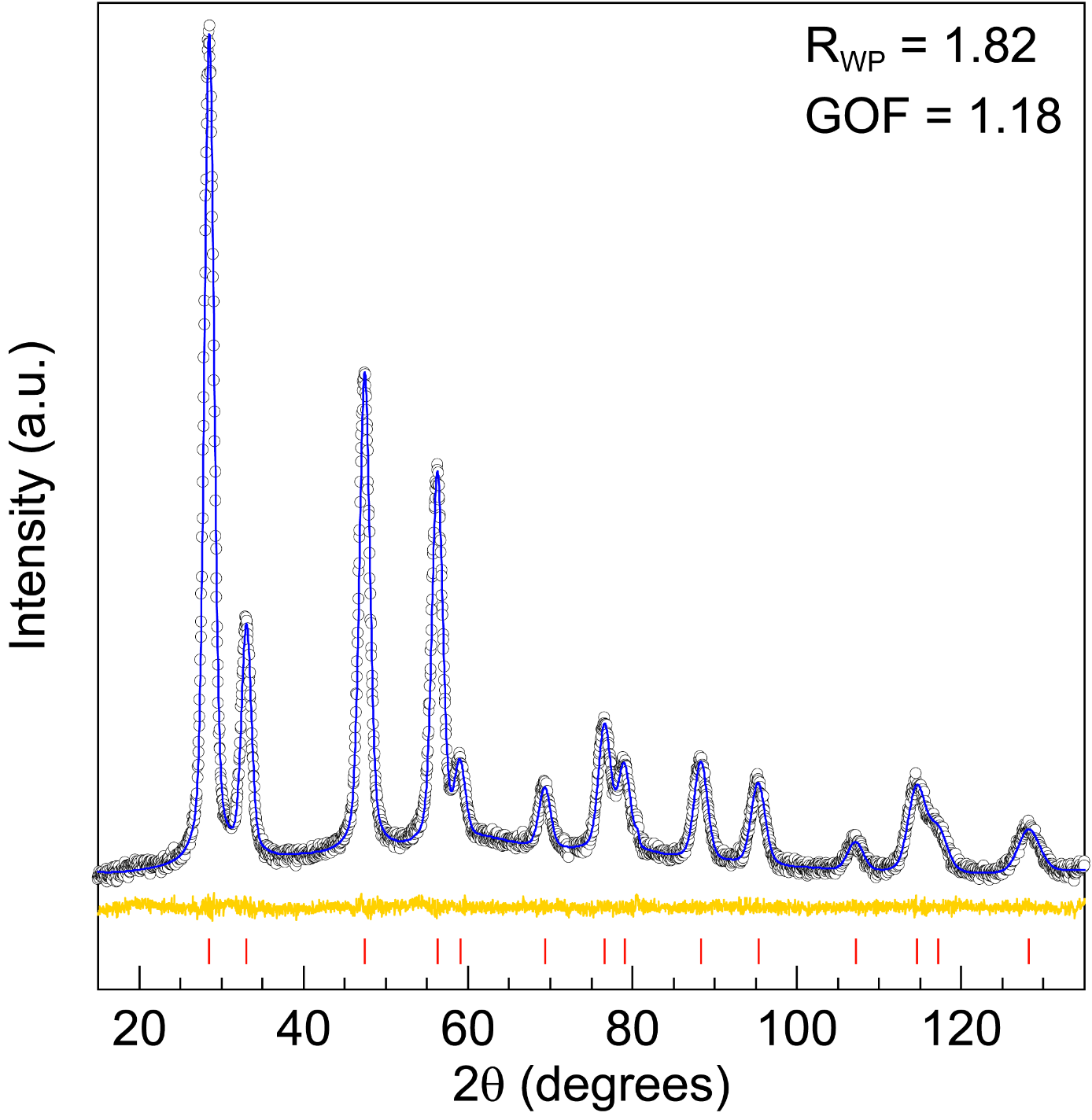


**Figure S2.** Experimental PXRD pattern (black open symbols) of CeO_2_ core NCs together with the corresponding Pawley refinement (blue solid line), difference curve (yellow solid line), and Bragg peaks’ positions (red vertical tick marks). The values of the weighted profile R factor (Rwp) and goodness-of-fit (GOF) are given to assess the quality of the refinement when combined with the visual inspection of the difference curve.


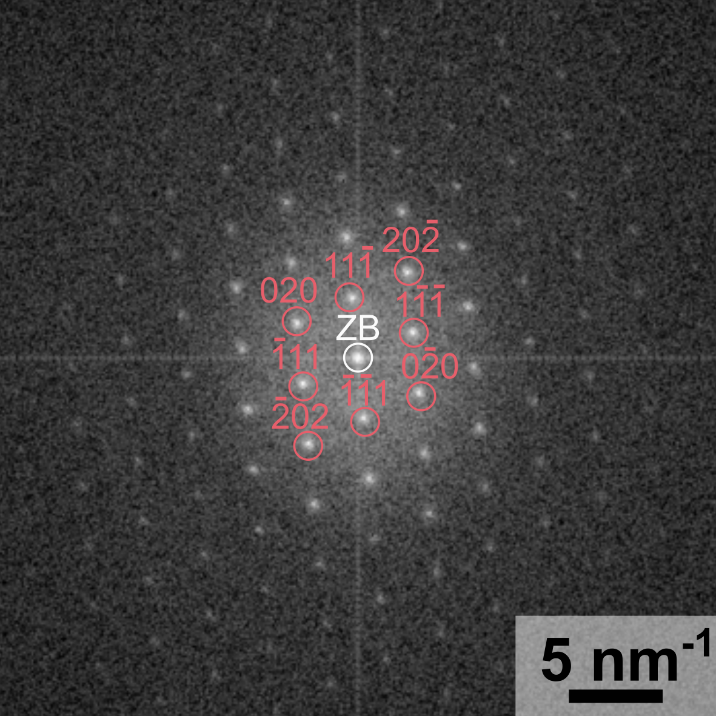


**Figure S3.** Fourier transform (FT) pattern of the HRTEM image of individual CeO_2_ core NC presented in the main text (**Figure 2b**). The FT pattern can be indexed with the calculated diffraction pattern of cubic CeO_2_ ($Fm\bar{3}m$) in the [101]-zone axis. For better readability of the FT pattern, indices of only a small number of reflections are reported. Indices of all other reflections can be deduced from the already indexed reflections and by symmetry. The zero-order beam (ZB) is marked with a white circle.


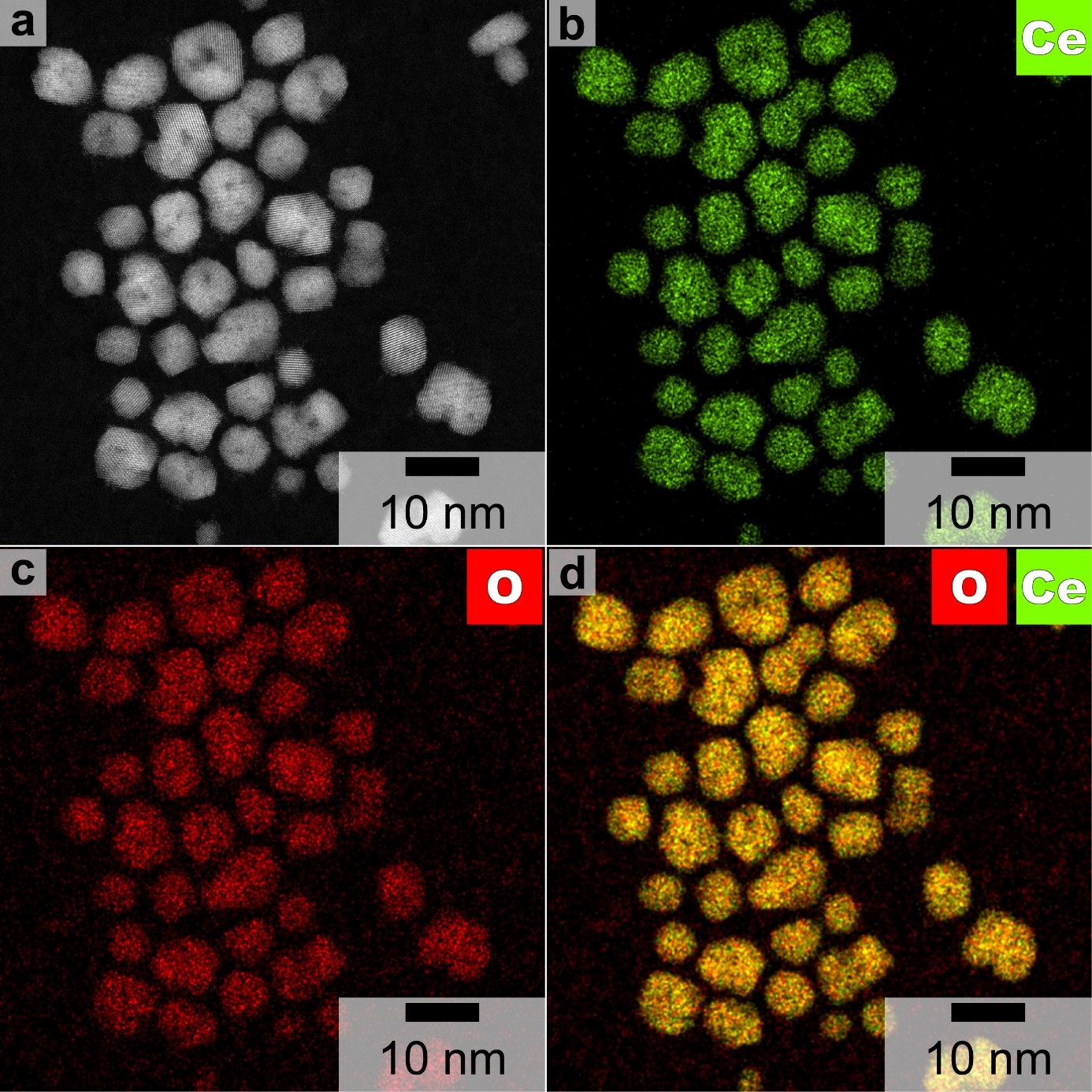


**Figure S4.** HAADF-STEM image (a) and EDX chemical maps of Ce (Lα line) (b), and O (Kα line) (c) together with their overlap (d) for CeO_2_ core NCs.


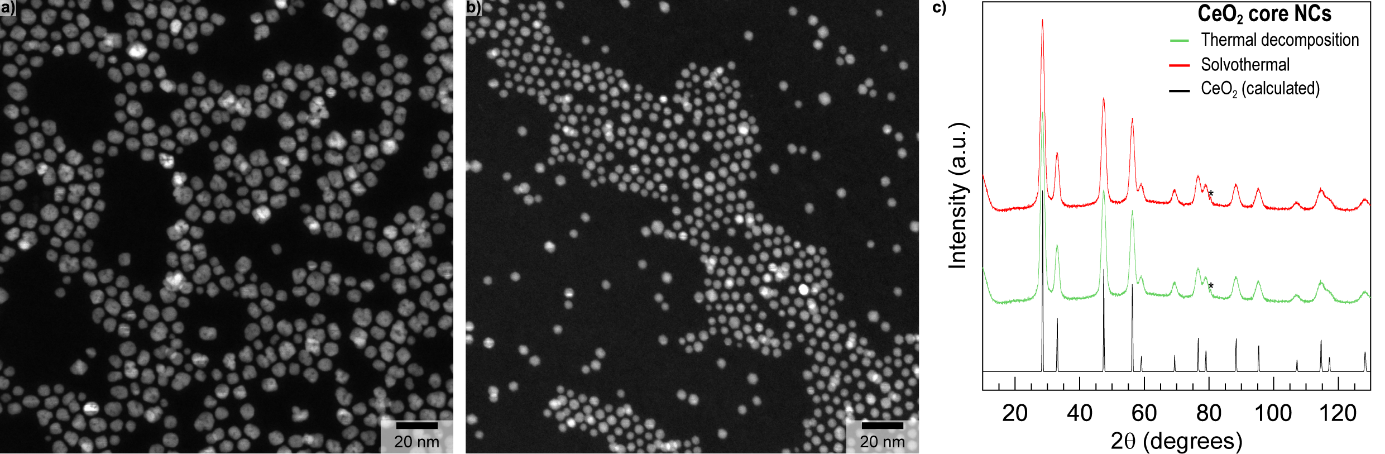


**Figure S5.** HAADF-STEM images of CeO_2_ core NCs synthesized by the thermal decomposition (a) and solvothermal (b) methods. Although larger CeO_2_ NCs can be synthesized by the thermal decomposition method (irregular shape, 7.4 ± 3.4 – 2σ standard deviation), the size and shape distributions are better controlled (spherical shape, 4.7 ± 1.4 – 2σ standard deviation) by the solvothermal method. The corresponding PXRD patterns of CeO_2_ core NCs synthesized by the thermal decomposition and solvothermal methods are in perfect agreement with the calculated reference PXRD pattern for microcrystalline CeO2 (c). The extra peak marked with a star (*i.e.* 2θ ≈ 81°) is due to the low-background (911)-oriented Si wafer that is used as a substrate onto which NCs are drop-casted.


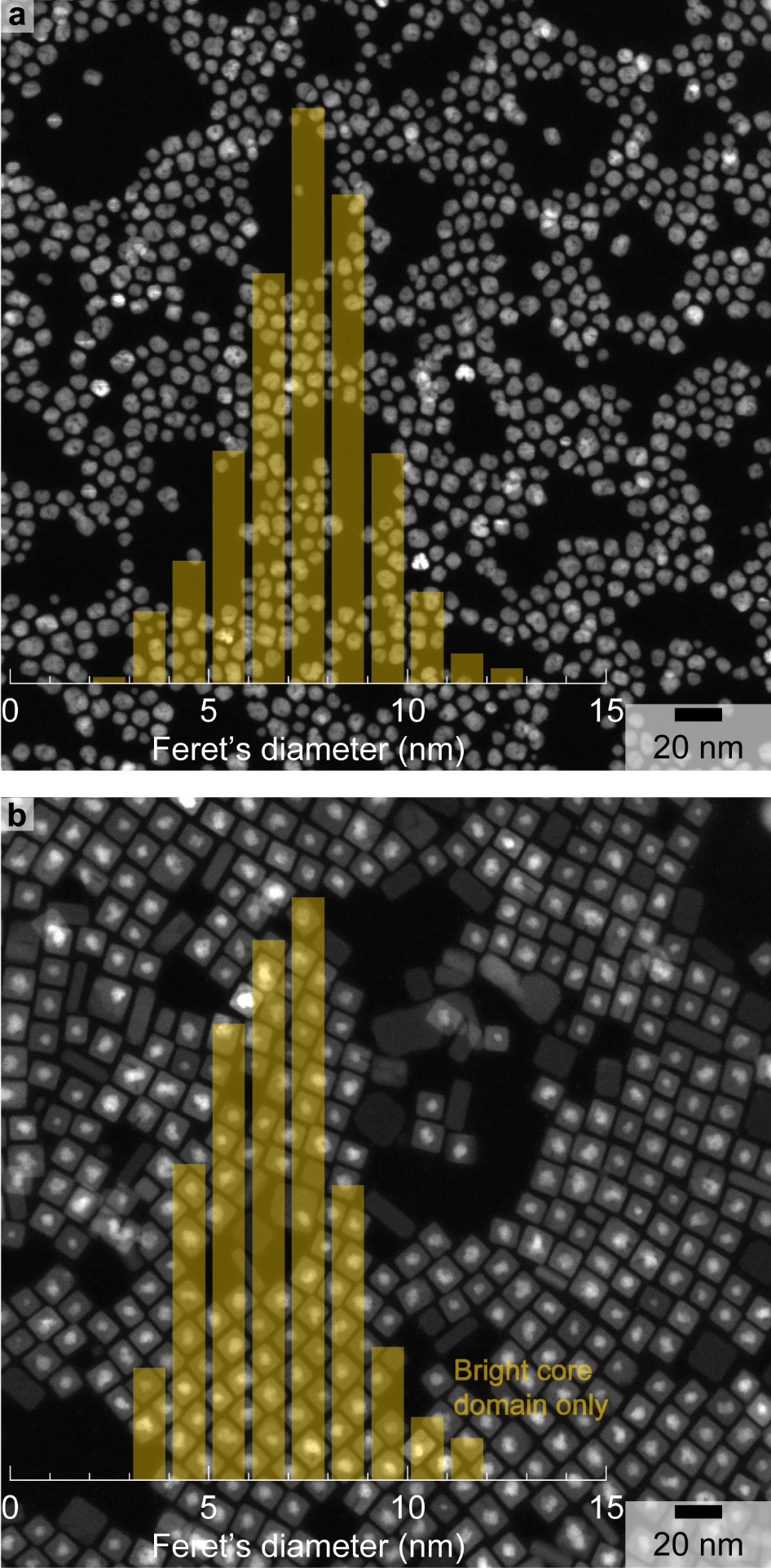


**Figure S6.** Low magnification HAADF-STEM images together with their corresponding overlaid size distribution histograms of CeO_2_ core (a) and CeO_2_/CaF_2_ core-shell (b) NCs. Size distribution histograms were obtained by measuring 1524 core (a) and 362 core-shell (b) individual NCs. Note that the size distribution histogram of CeO_2_/CaF_2_ NCs refers to the core region alone (bright regions on the corresponding HAADF-STEM image). The comparison of the size distribution histograms of the core domains before (a) and after (b) shell deposition shows a slight decrease of the size.


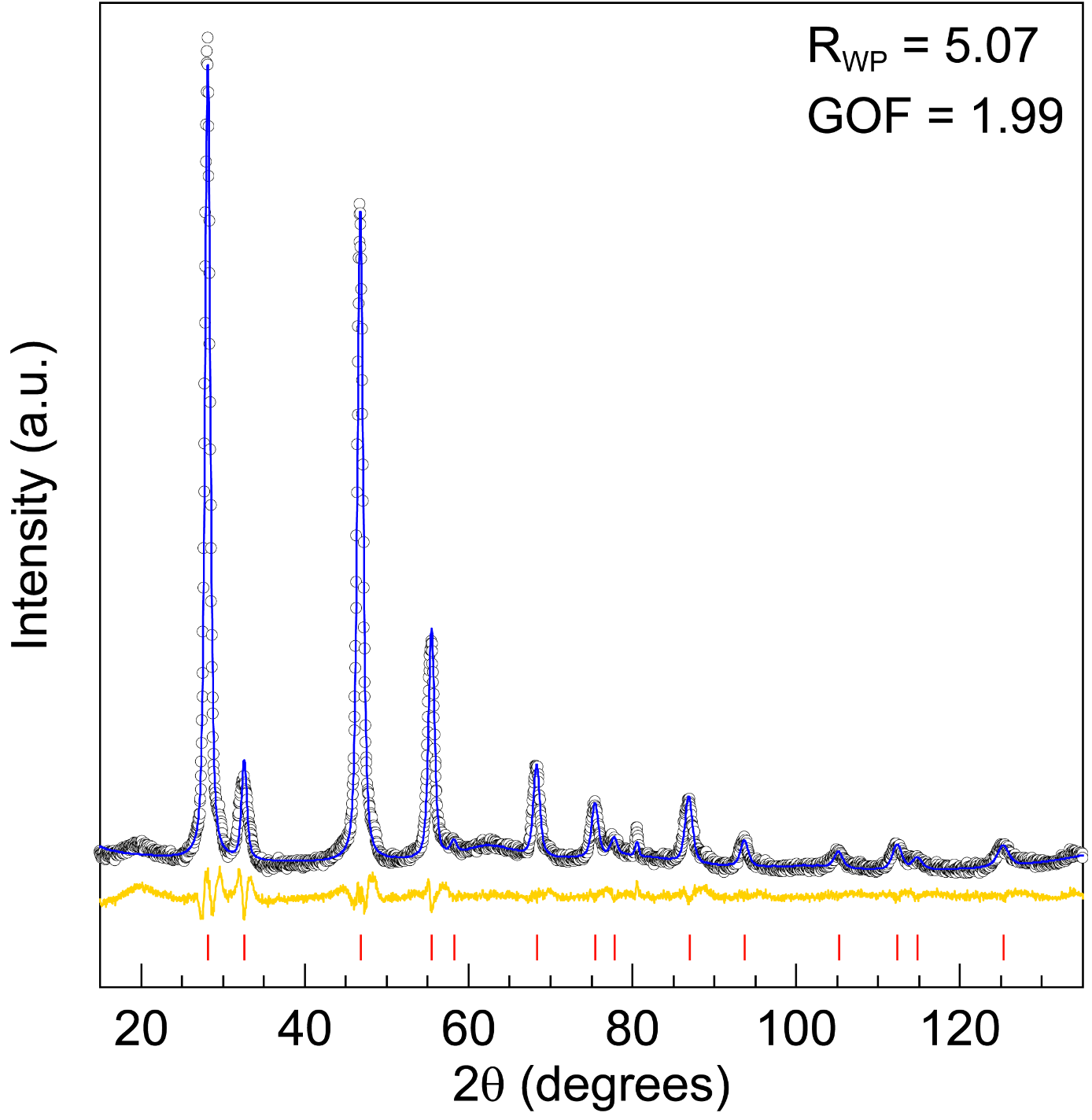


**Figure S7.** Experimental PXRD pattern (black open symbols) of CeO_2_/CaF_2_ core-shell NCs together with the corresponding Pawley refinement (blue solid line), difference curve (yellow solid line), and Bragg peaks’ positions (red vertical tick marks). The values of the weighted profile R factor (Rwp) and goodness-of-fit (GOF) are given to assess the quality of the refinement when combined with the visual inspection of the difference curve.


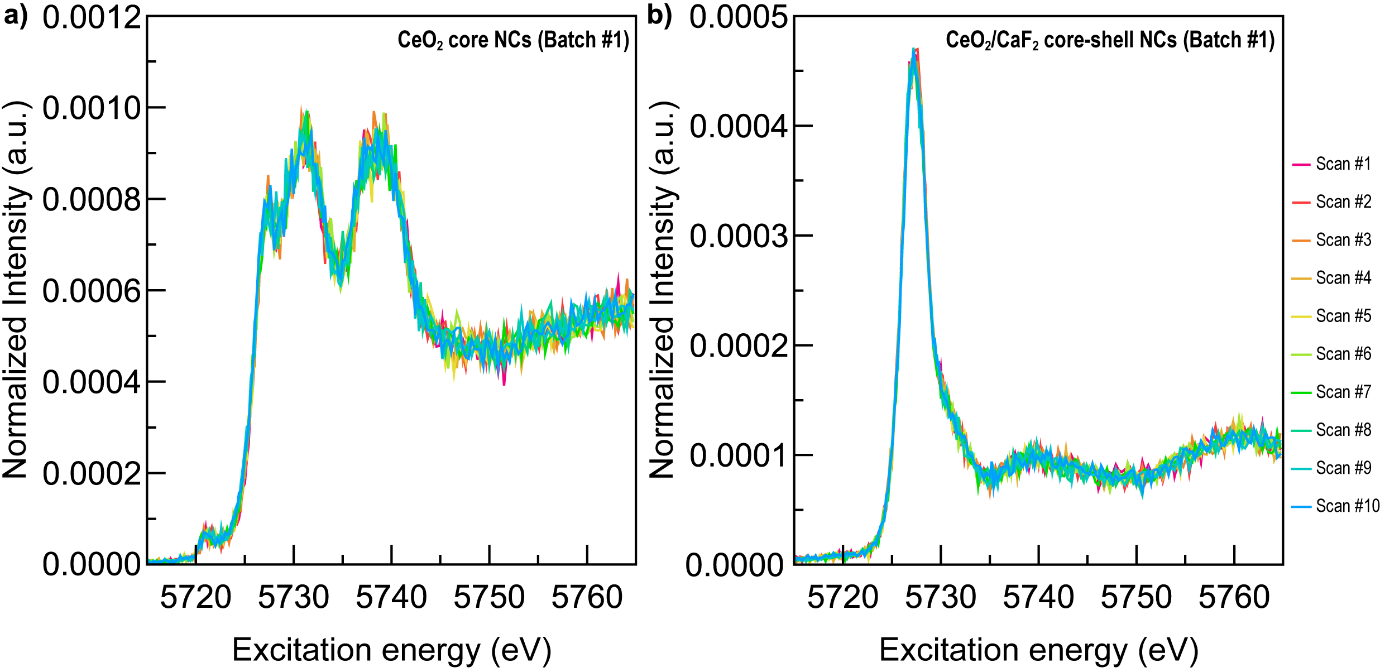


**Figure S8.** Radiation beam damage testing at the Ce L_3_-edge of the CeO_2_ core (a) and CeO_2_/CaF_2_ core-shell (b) NCs. No radiation beam damage is observed. All scans were averaged, normalized, and energy calibrated to yield the final spectra.


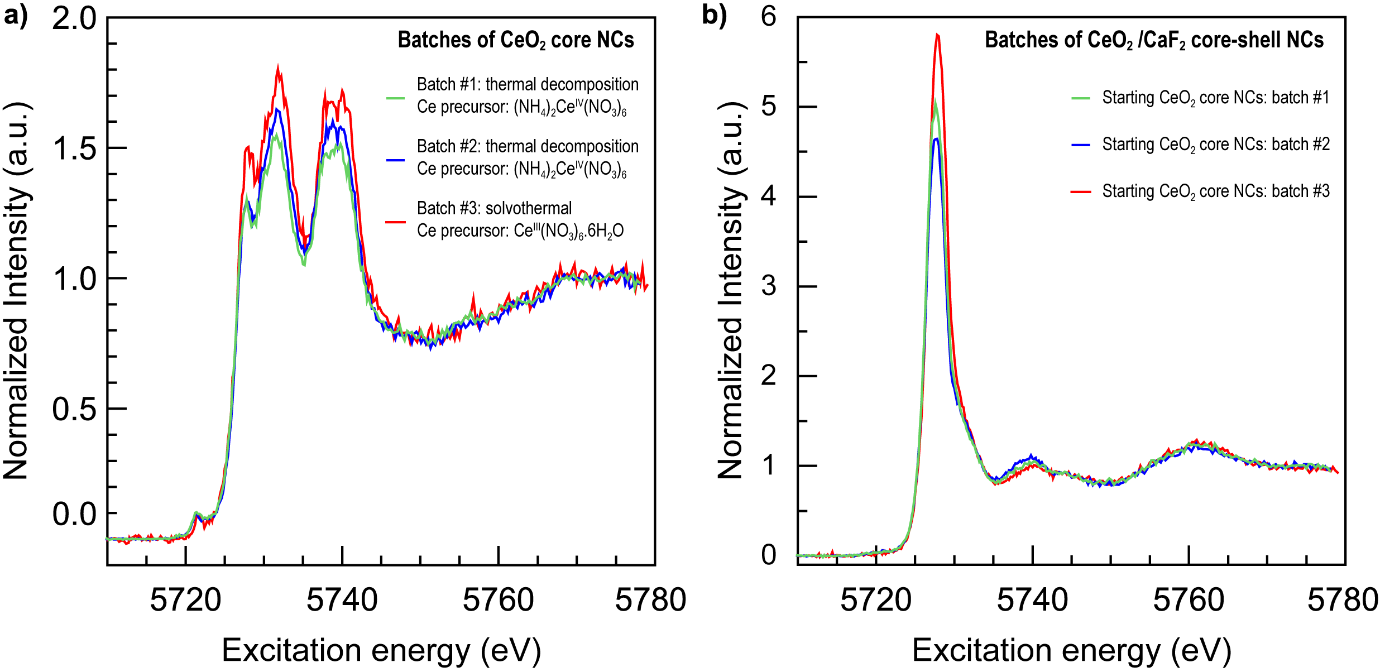


**Figure S9.** Comparison of the Ce L_3_-edge HR-XANES spectra for three different batches of CeO_2_ core (a) and CeO_2_/CaF_2_ core-shell (b) NCs. For “Batch #1”, CeO_2_ core NCs were synthesized by the thermal decomposition method (*i.e.* (NH_4_)_2_Ce^IV^(NO_3_)_6_ precursor) and immediately used (*i.e.* the day after) for CaF_2_ shell growth. For “Batch #2”, CeO_2_ core NCs were synthesized by the exact same synthesis method as for “Batch #1” but aged for two months (room temperature, air) before growing the CaF_2_ shell. Finally, for “Batch #3”, ultra-small (4.7 ± 1.4 nm – 2σ standard deviation) were synthesized by the solvothermal method (*i.e.* Ce^III^(NO_3_)_3_•6H_2_O) and immediately used for CaF_2_ shell growth. No changes in oxidation state purity are observed between the three different batches, thus indicating that neither the size of the starting CeO_2_ core NCs nor the synthesis method or the starting oxidation state of the Ce precursor seem to influence the final oxidation state.


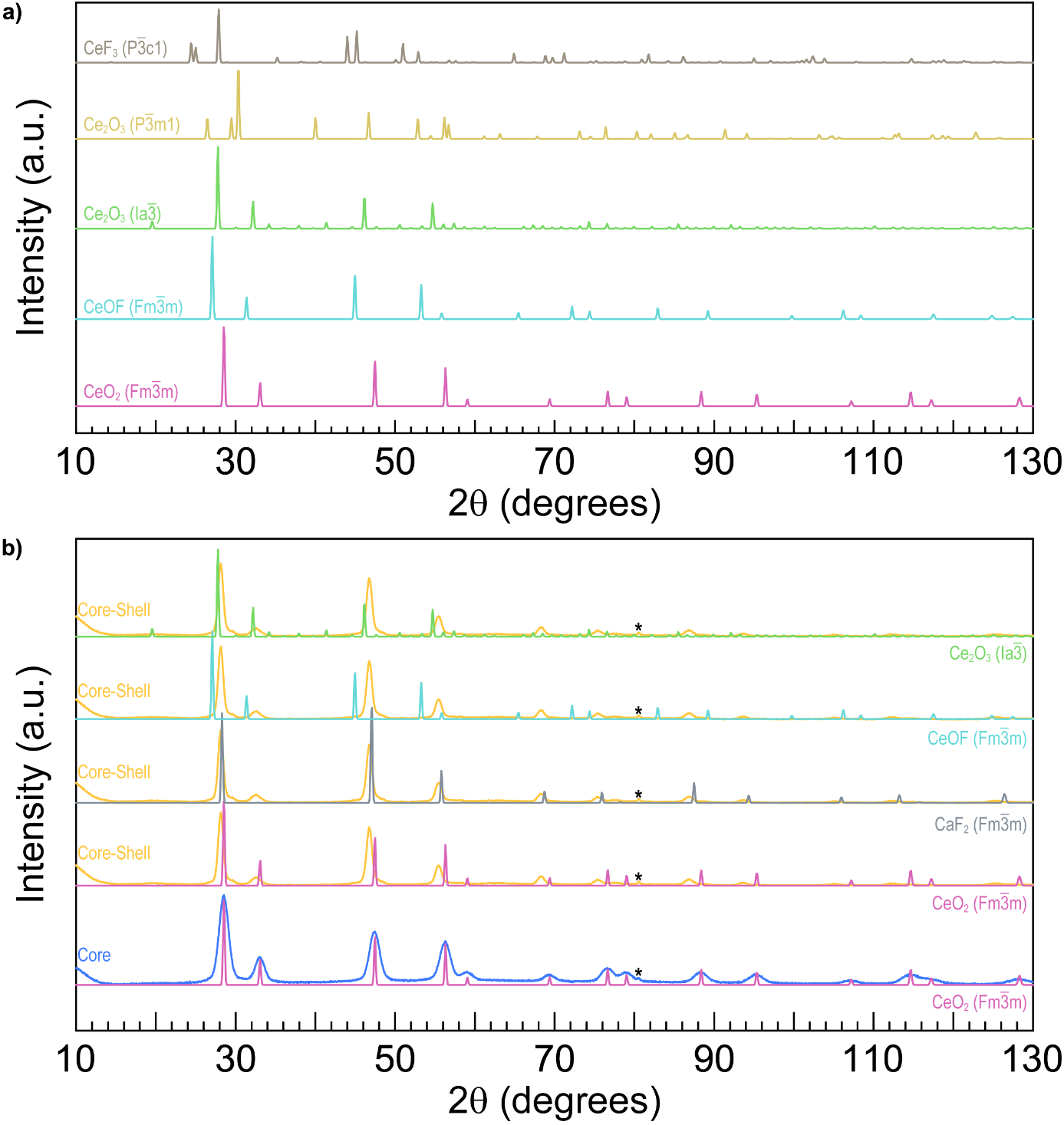


**Figure S10.** (a) Theoretical PXRD patterns (copper radiation) for various Ce-based crystalline materials with different Ce oxidation states (III or IV) and chemical compositions (oxide, fluoride, oxyfluoride). (b) Comparison of experimental PXRD patterns of CeO_2_ core (blue solid line) and CeO_2_/CaF_2_ core-shell (orange solid line) NCs with probable cubic candidates. The extra peak marked with a star (*i.e.* 2θ ≈ 81°) is due to the low-background (911)-oriented Si wafer that is used as a substrate onto which NCs are drop-casted.

The formation of trigonal cerium trifluoride (CeF_3_) and trigonal cerium sesquioxide (Ce_2_O_3_) can be excluded because their corresponding Bragg peaks positions do not match with the experimental PXRD patterns. Although the best agreement for the experimental pattern of CeO_2_/CaF_2_ core-shell NCs is with CaF_2_, the co-existence of other cubic phases in various proportions including CeO_2_, Ce_2_O_3_, CeOF cannot be excluded especially if lattice parameters are different from those observed for microcrystalline references. Additionally, the formation of complex solid solutions with partially intermixed cation and/or anion networks cannot be excluded. Unfortunately, PXRD cannot be used to differentiate between the different possibilities.


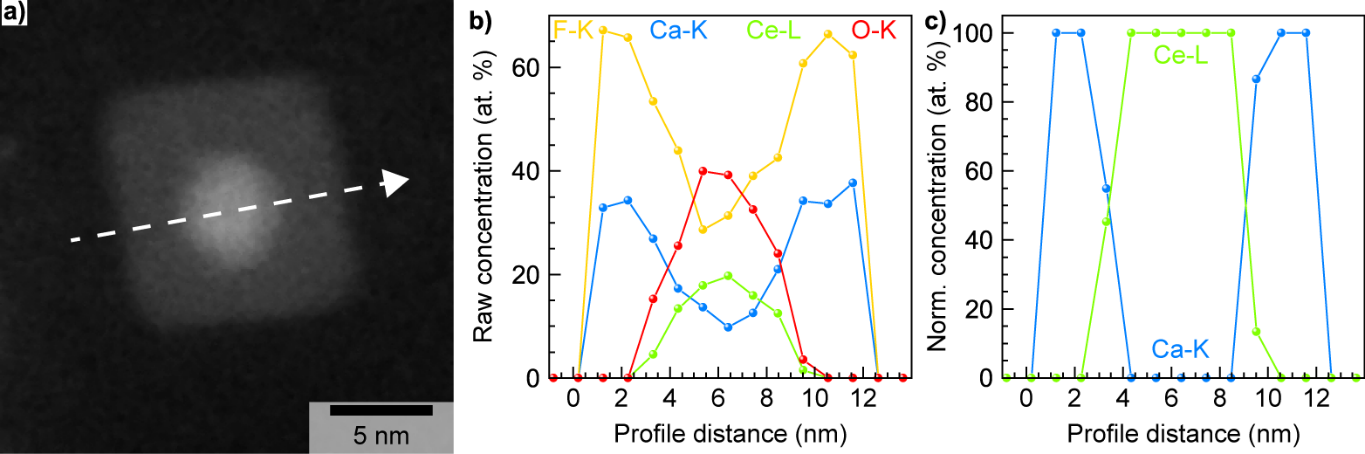


**Figure S11.** HAADF-STEM image (a), normalized raw (b) and calculated (applying the subshell approach to raw elemental concentrations - c) chemical profiles obtained after the quantification of the experimental EDX line-scan (white arrow in a) of an individual CeO_2_/CaF_2_ core-shell NC.


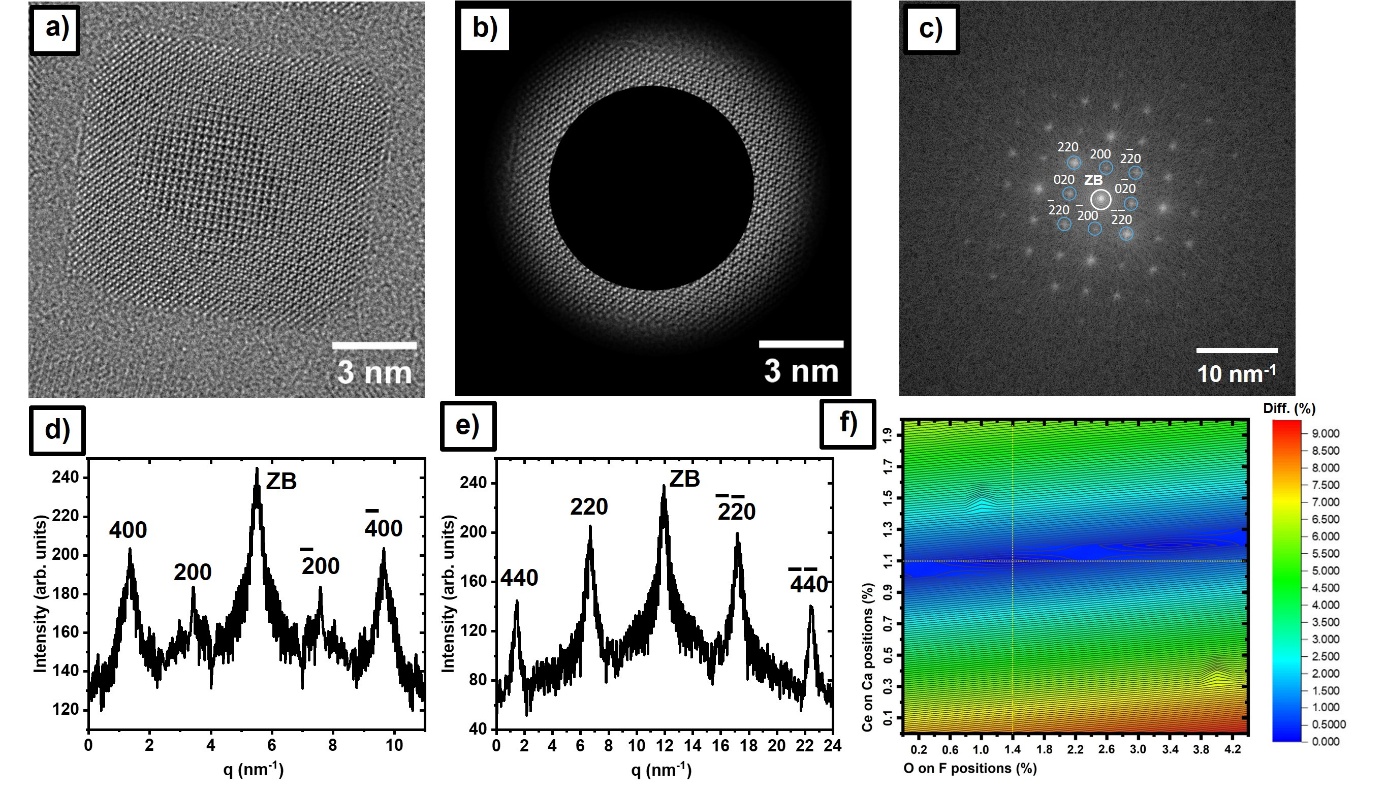


**Figure S12.** HRTEM images of a single CeO_2_/CaF_2_ core-shell NC (a) and a thin (*ca.* 2 nm) CaF_2_ shell region expected to contain about 1 at.% Ce and 2 at.% O from EDX line scan analysis (b). Corresponding FT pattern (c) of the HRTEM image (b) together with the integrated intensities of the (hkl) reflections marked in the FT pattern (c). Discrepancies are observed between the intensity distribution of the experimental FT pattern of the CaF_2_ shell (d-e) and the calculated intensity of the CaF_2_. Particularly, the (200) intensity (sensitive to the substitution of Ca by Ce and/or the substitution of F by O) in the FT pattern of the shell is 8% higher compared to the one expected for CaF_2_. The discrepancy is reduced to 0.1% by considering a 1.1 at.% substitution of Ca by Ce and a 2.8 at.% substitution of F by O on the cation and anion sites (f), in relatively good agreement with EDX data analysis.


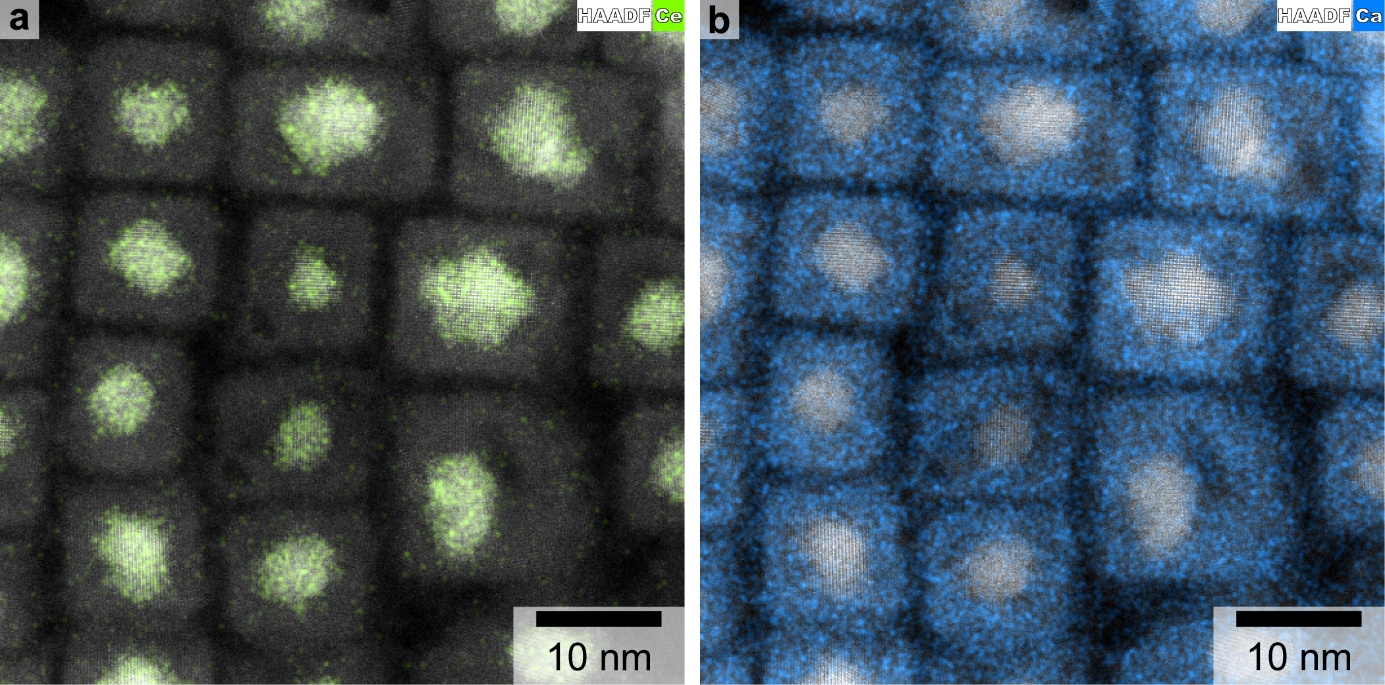


**Figure S13.** Overlapped HAADF-STEM image with the corresponding EDX elemental map of Ce (a) and Ca (b) for CeO_2_/CaF_2_ core-shell NCs.


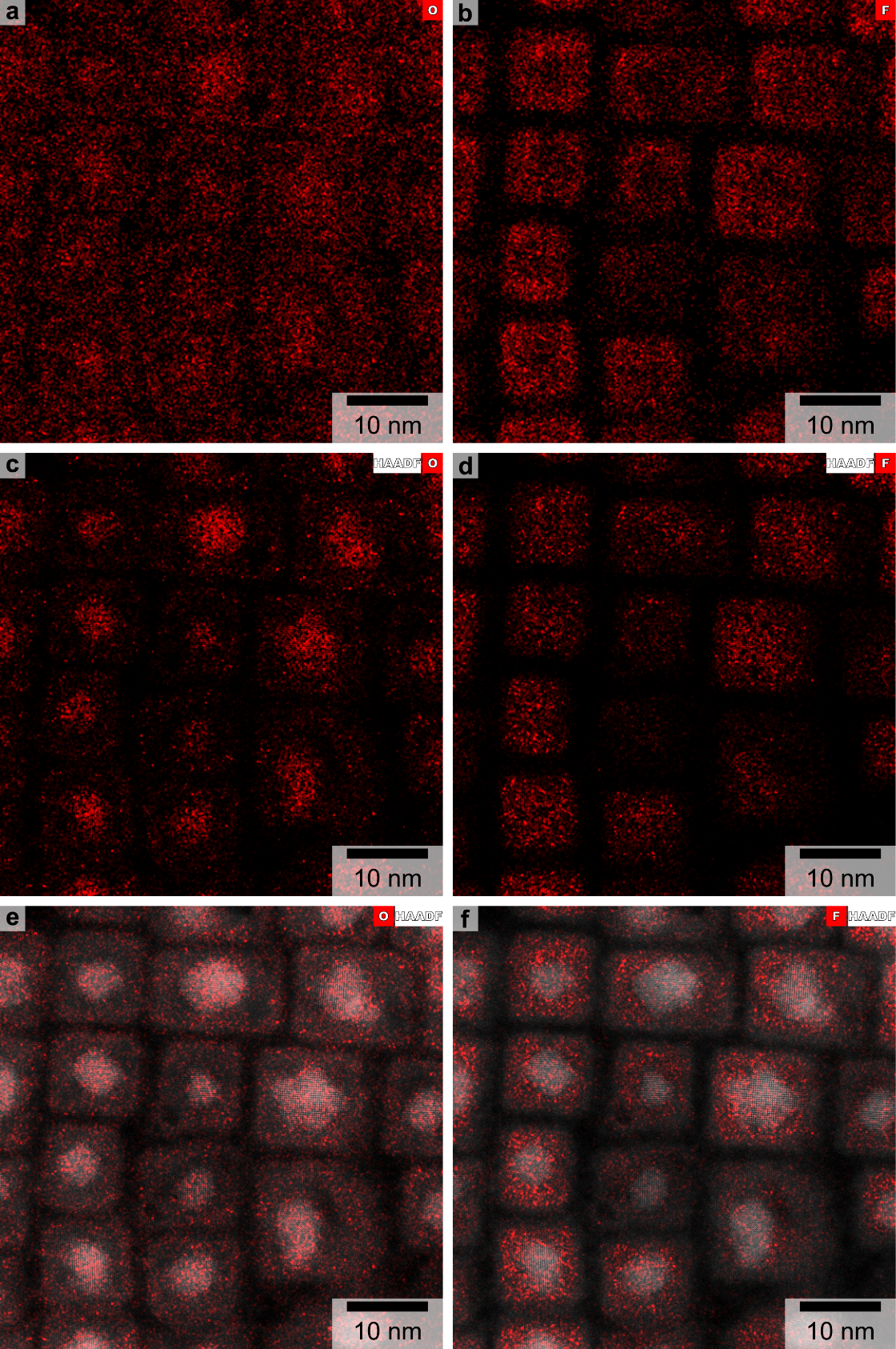


**Figure S14.** EDX elemental maps of Ce (a) and F (b) revealing differences regarding the spatial distribution of O and F within the particles’ boundaries. As differences are not easily noticeable, blending with the corresponding HAADF-STEM image has been implemented in Adobe Illustrator by using different blending modes. c) Overlapped raw O EDX map (back image – 100% opacity) and HAADF-STEM image (front image – 100% opacity). In Adobe Illustrator, the “Overlay” blending mode was applied to the front HAADF-STEM image. d) Overlapped raw F EDX map (back image – 100% opacity) and HAADF-STEM image (front image – 100% opacity). In Adobe Illustrator, the “Overlay” blending mode was applied to the front HAADF-STEM image. e) Overlapped HAADF-STEM image (back image – 60% opacity) and raw O EDX map (front image – 100% opacity). In Adobe Illustrator, the “Color Dodge” blending mode was applied to the front raw O EDX map. f) Overlapped HAADF-STEM image (back image – 60% opacity) and raw F EDX map (front image – 100% opacity). In Adobe Illustrator, the “Color Dodge” blending mode was applied to the front raw F EDX map.


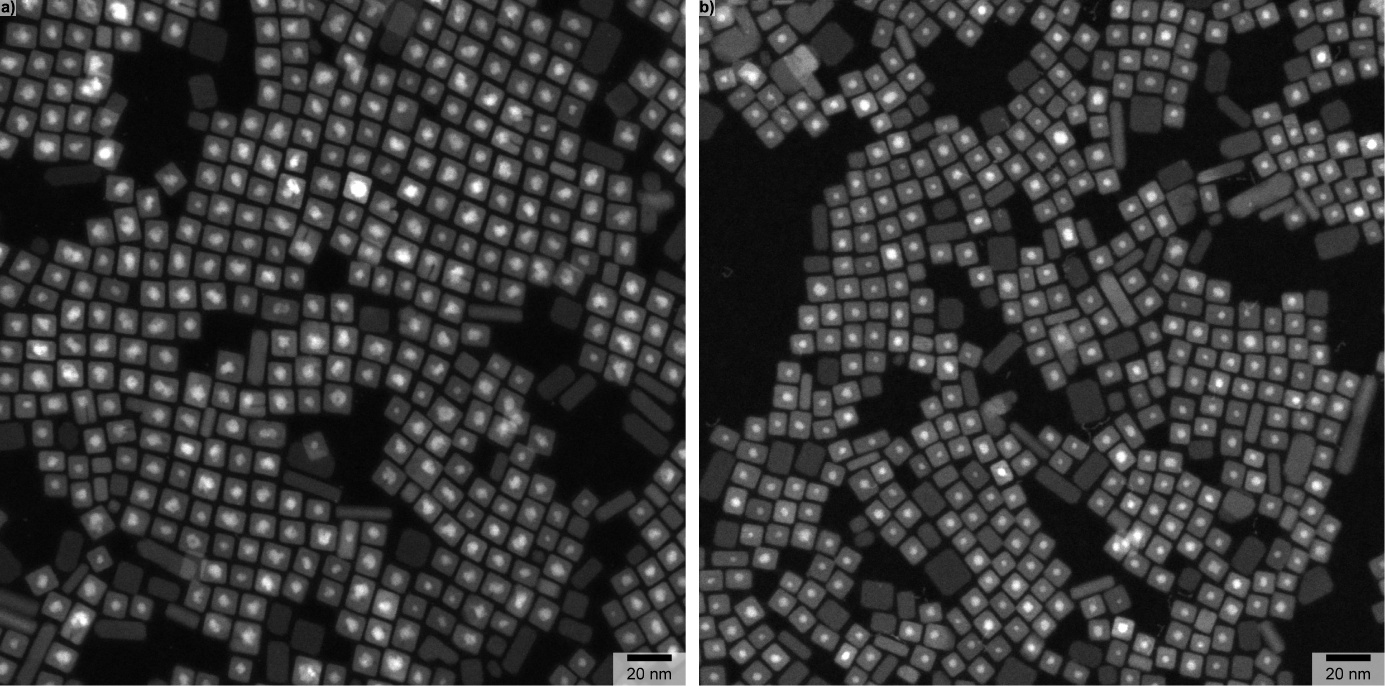


**Figure S15.** HAADF-STEM images of CeO_2_/CaF_2_ core-shell NCs synthesized by the exact same synthesis method but using CeO_2_ core NCs synthesized by the thermal decomposition (a) and solvothermal (b) methods.


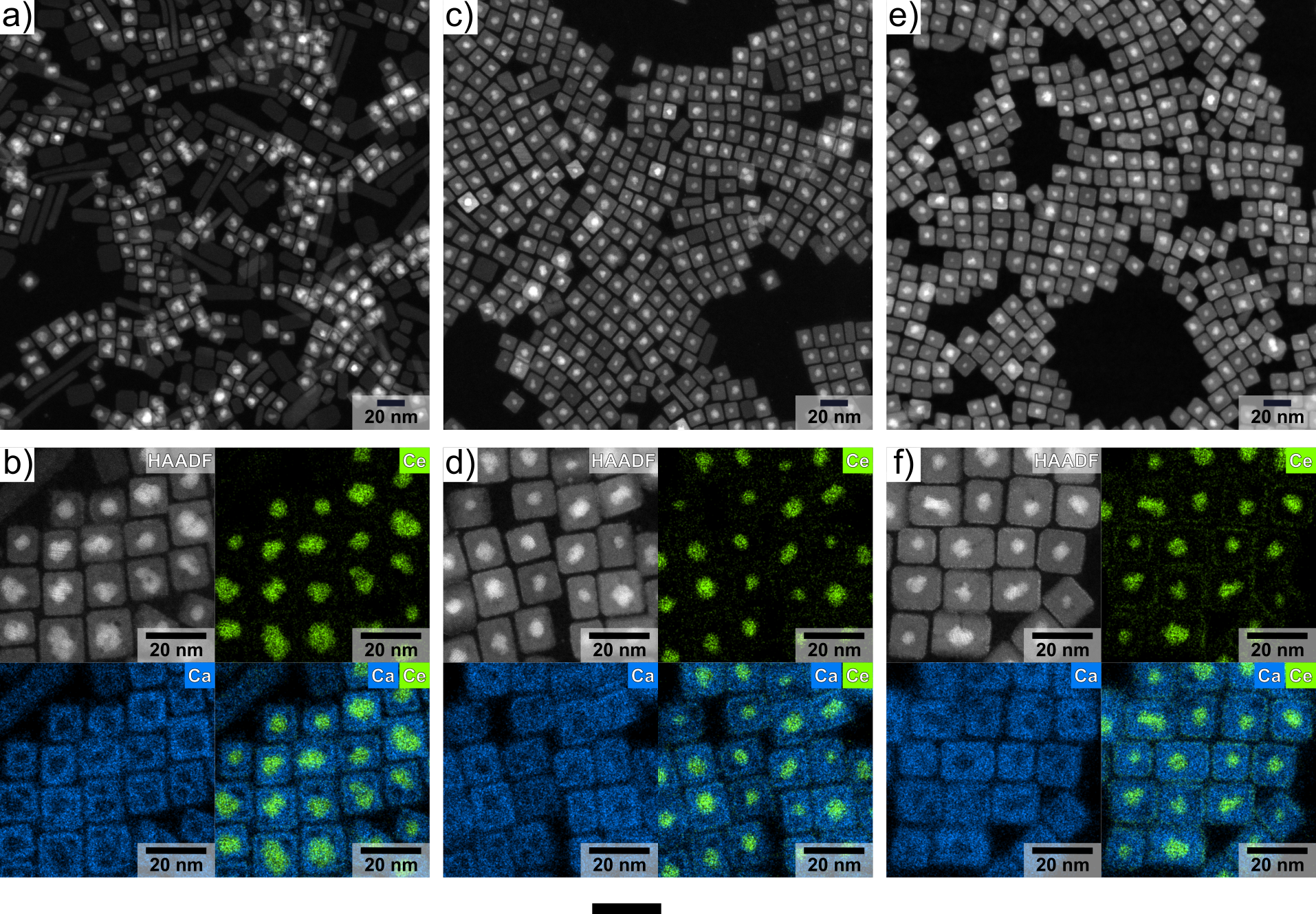


**Figure S16.** Low magnification HAADF-STEM images of CeO_2_/CaF_2_ core-shell NCs synthesized by using 5 mmol (a), 11 mmol (c), and 22 mmol (e) of oleic acid (OA) while using the same starting CeO_2_ core NCs and the exact same quantity of calcium trifluoroacetate (1 mmol). For each synthesis, HAADF-STEM images and their corresponding EDX elements of Ce (L line) and Ca (K line) are shown together with their overlap (b, d, f).


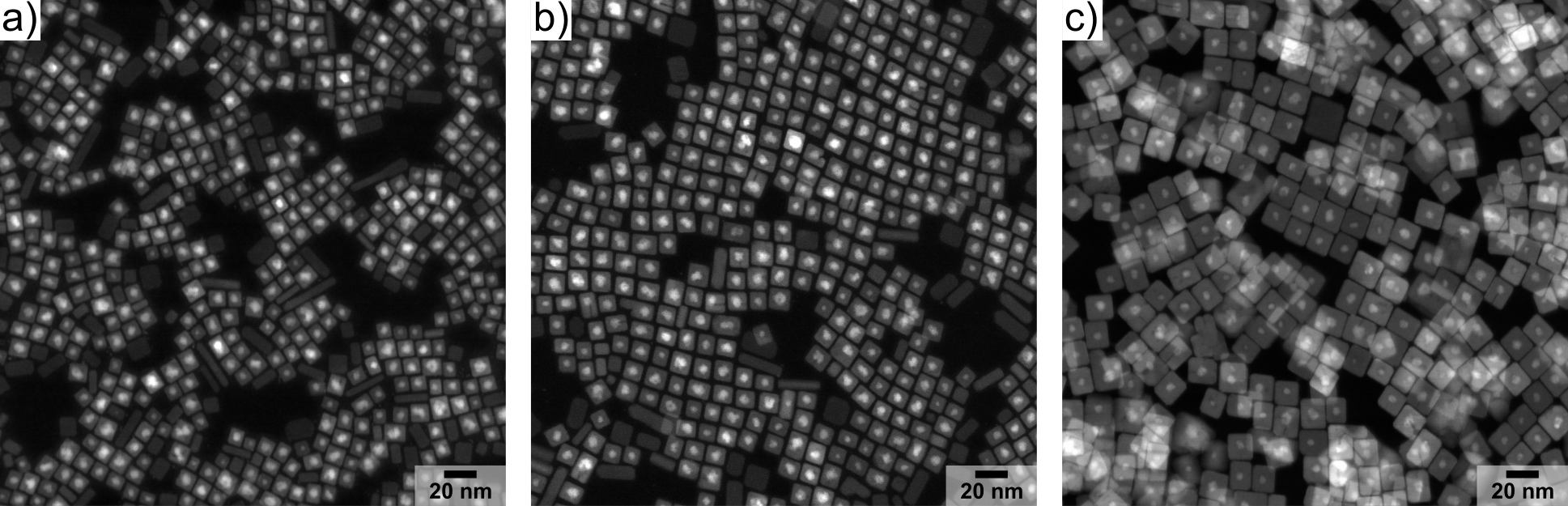


**Figure S17.** Low magnification HAADF-STEM images of CeO_2_/CaF_2_ core-shell NCs synthesized by using 0.5 mmol (a), 1 mmol (b), and 2 mmol (c) of calcium trifluoroacetate while using the same starting CeO_2_ core NCs and keeping the oleic acid (OA):Ca molar ratio constant (11).


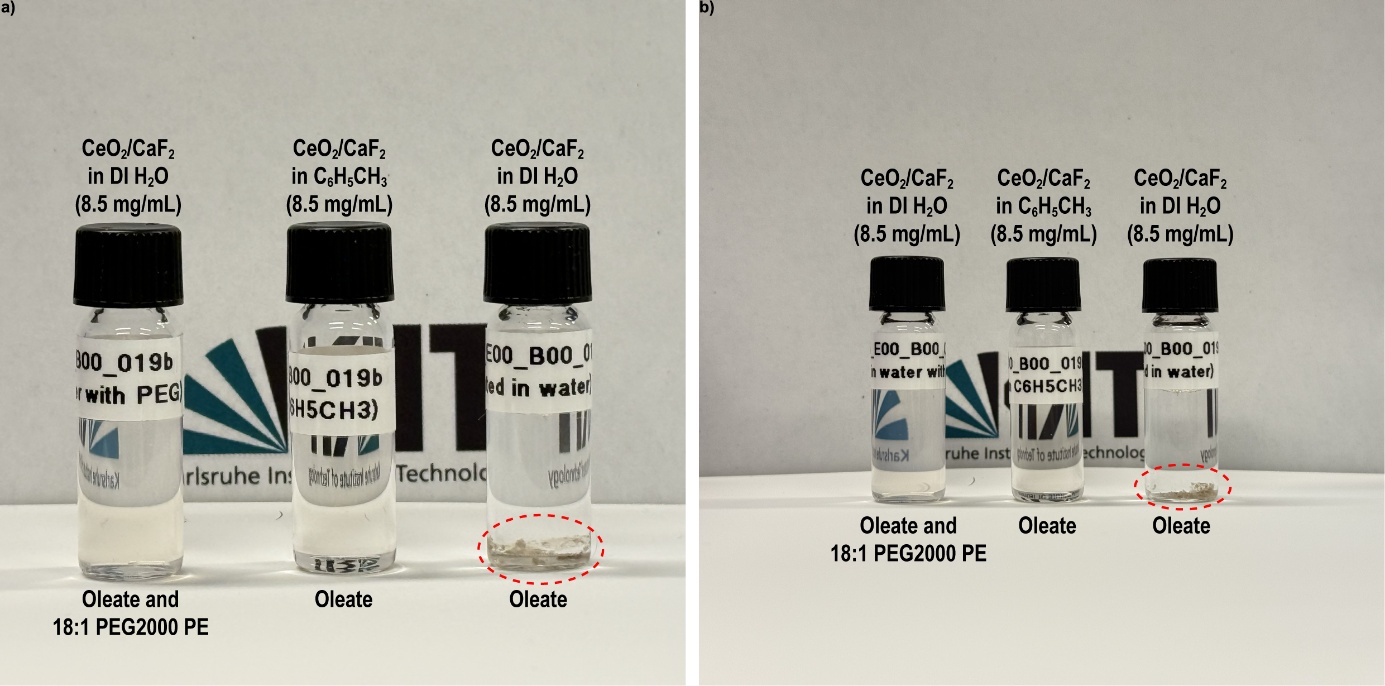


**Figure S18.** Digital photographs comparing the dispersion of as-synthesized (i.e. capped with oleate ligands) and surface functionalized (18:1 PEG2000 PE phospholipids) CeO2/CaF2 core-shell NCs in different solvents (C_6_H_5_CH_3_ and DI water) immediately after dispersion (a) and after 12h (b). The as-synthesized CeO_2_/CaF_2_ core-shell NCs are easily dispersible in non-polar organic solvents such as for instance C_6_H_5_CH_3_ (middle glass vial in panels a) and b)) but immediately precipitate if introduced in DI water (right glass vial in panels a) and b)). The utilization of phospholipids (18:1 PEG2000 PE) can be used to form stable colloidal suspension of CeO_2_/CaF_2_ core-shell NCs in DI water (left glass vial in panels a) and b)). With 18:1 PEG2000 PE phospholipids, a stable colloidal suspension of CeO_2_/CaF_2_ core-shell NCs in DI water was observed for at least 10 days.


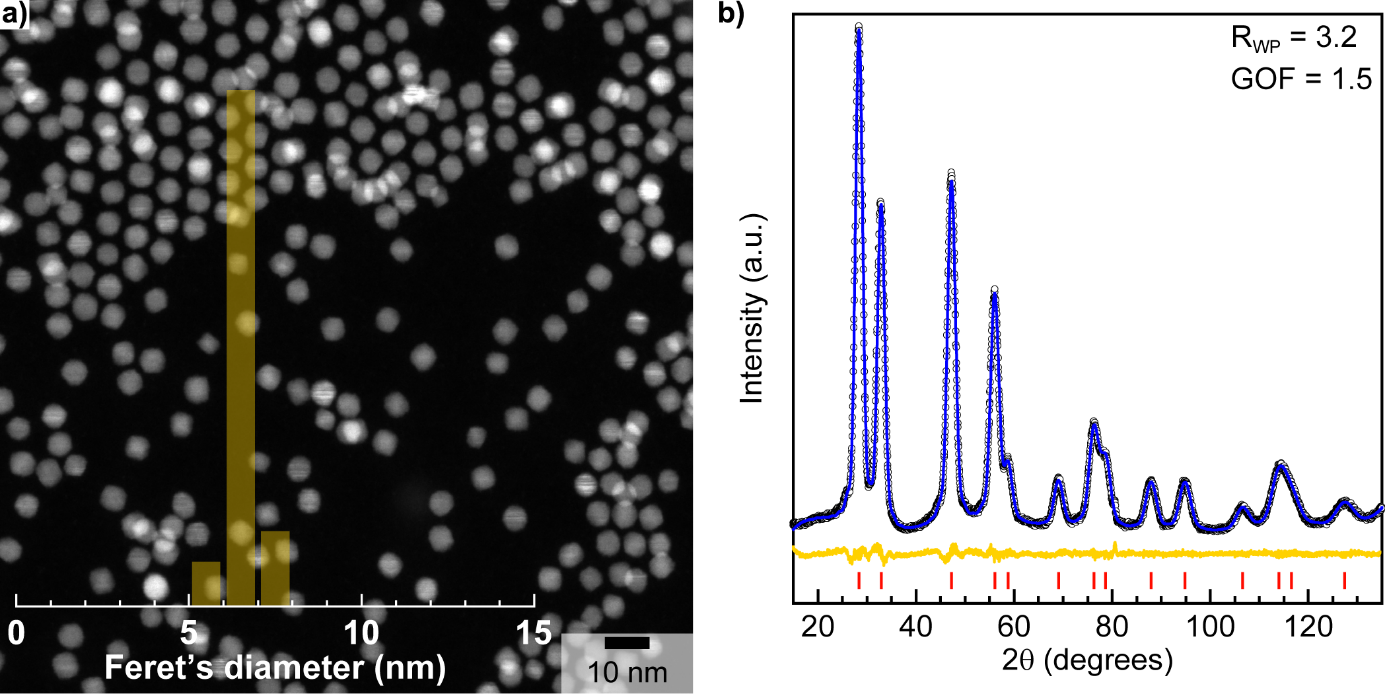


**Figure S19.** Low magnification HAADF-STEM image together with the corresponding overlaid size distribution histogram (a) and PXRD pattern (b) of UO_2_ core NCs. The experimental PXRD pattern (black open symbols) of UO_2_ core NCs is shown together with the corresponding Pawley refinement (blue solid line), difference curve (yellow solid line), and Bragg peaks’ positions (red vertical tick marks). The values of the weighted profile R factor (Rwp) and goodness-of-fit (GOF) are given to assess the quality of the refinement when combined with the visual inspection of the difference curve.


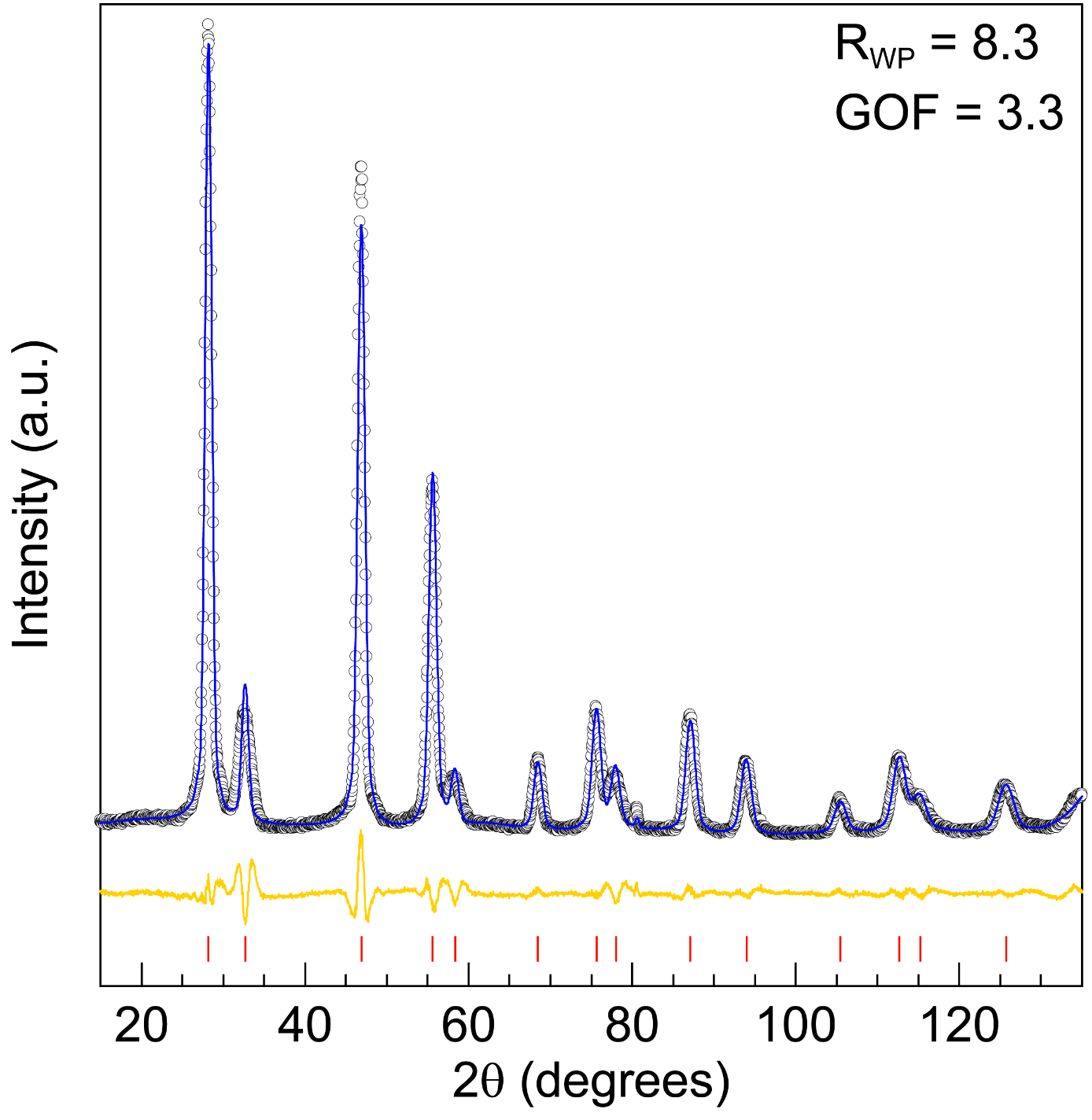


**Figure S20.** Experimental PXRD pattern (black open symbols) of UO_2_/CaF_2_ core-shell NCs together with the corresponding Pawley refinement (blue solid line), difference curve (yellow solid line), and Bragg peaks’ positions (red vertical tick marks). The values of the weighted profile R factor (Rwp) and goodness-of-fit (GOF) are given to assess the quality of the refinement when combined with the visual inspection of the difference curve.


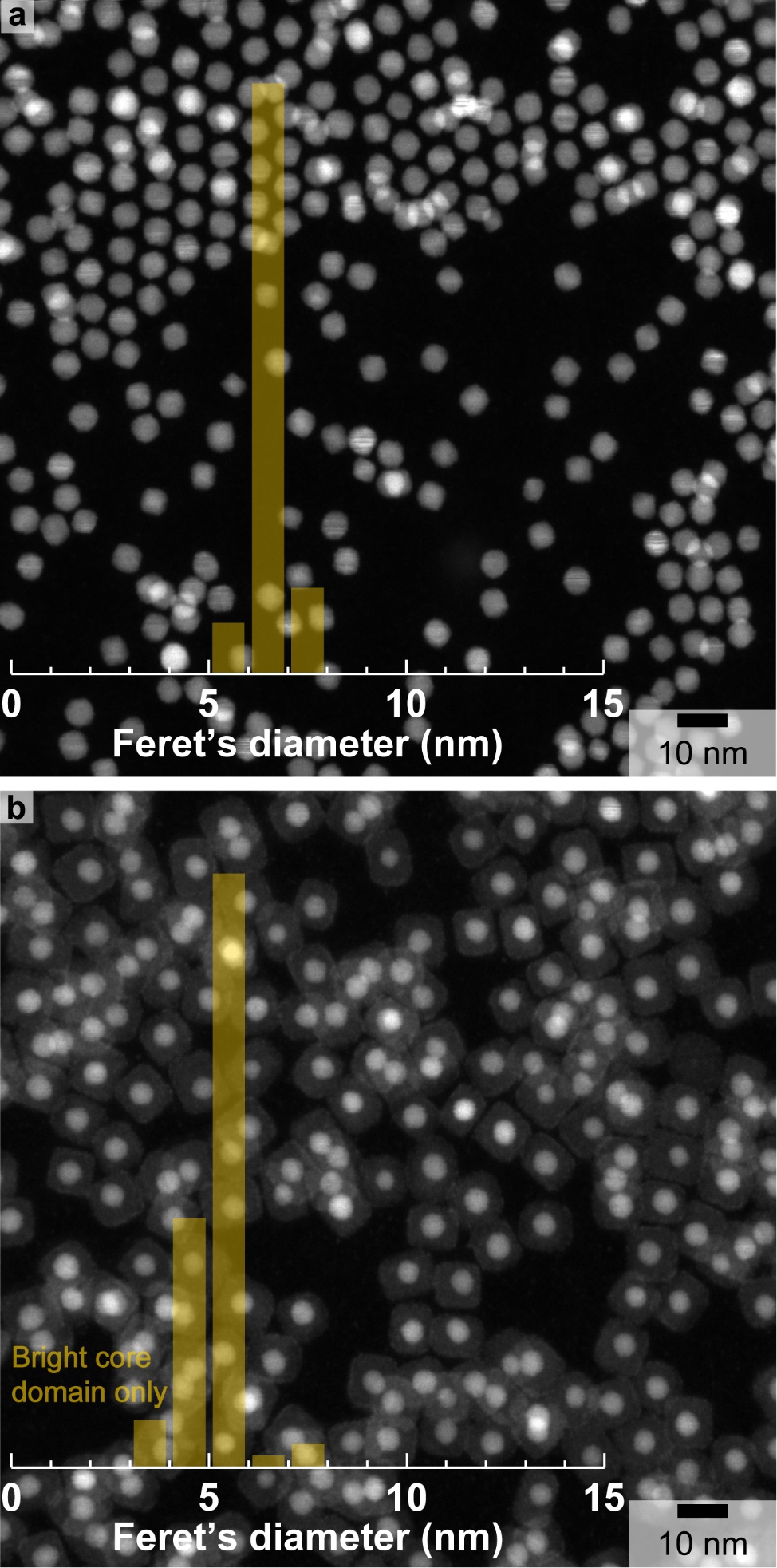


**Figure S21.** Low magnification HAADF-STEM images together with their corresponding overlaid size distribution histograms of UO_2_ core (a) and UO_2_/CaF_2_ core-shell (b) NCs. Note that the size distribution histogram of UO_2_/CaF_2_ NCs refers to the core region alone (bright regions on the corresponding HAADF-STEM image). The comparison of the size distribution histograms of the core domains before (a) and after (b) shell deposition shows a non-negligible decrease of the size.


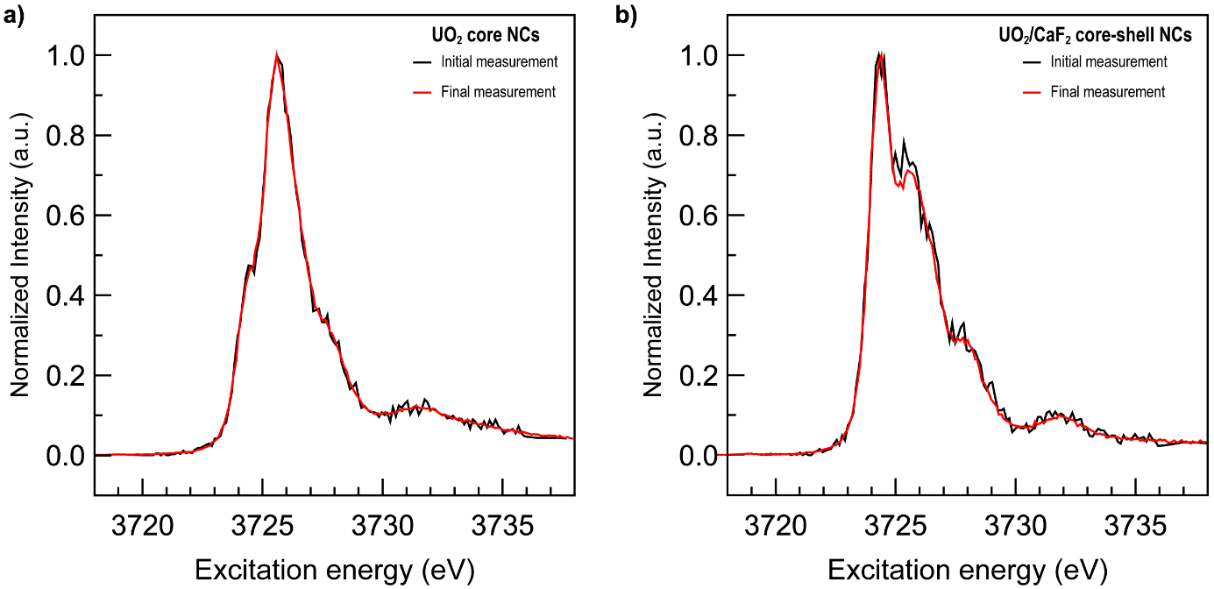


**Figure S22.** Initial (black) and final (red) measurements showing radiation beam damage testing at the U M_4_-edge HR-XANES for UO_2_ (a) and UO_2_/CaF_2_ core-shell (b) NCs. No radiation damage is observed.


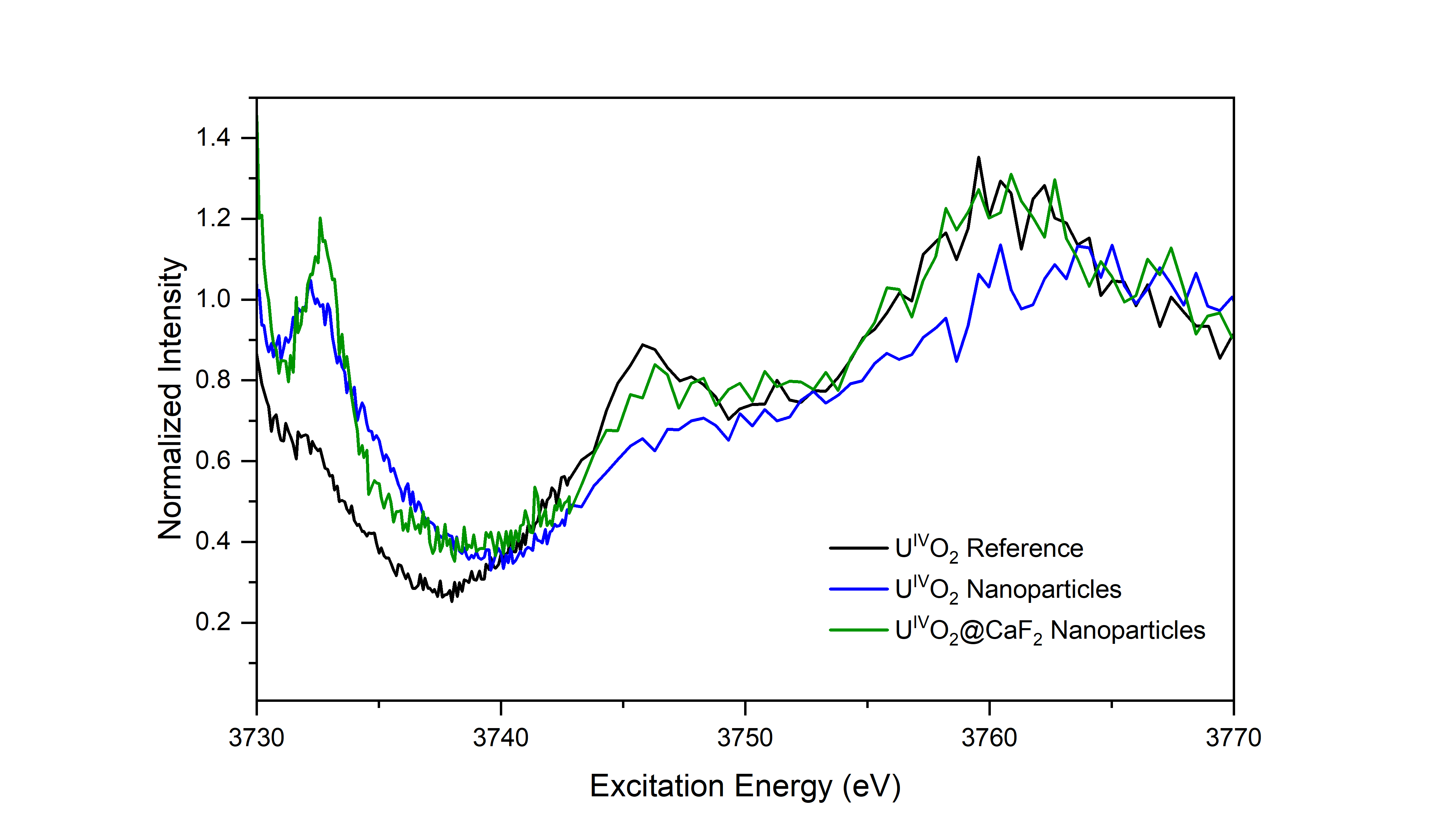


**Figure S23.** U M_4_-edge HR-XANES post-edge region of microcrystalline bulk-like UO_2_ (reference, black), UO_2_ core NCs (blue), and UO_2_/CaF_2_ core-shell NCs (green).

Weaker features at 3728.8 and 3732.6 eV on the HR-XANES spectrum of UO_2_/CaF_2_ core-shell NCs (Main text – Figure 4f) are indicative of the presence of the uranyl cation (U^VI^O_2_^2+^) cation. To investigate whether the uranyl cation is integrated into the UO_2_/CaF_2_ core-shell structure, the post-edge region is analyzed. The features around 3746 and 3760 eV are characteristic of the cubic structure of bulk UO_2_, and changes in these peaks report on the deviations from cubic behavior in UO_2_ systems. Inspection of these peaks shows much greater similarity between bulk UO_2_ and UO_2_/CaF_2_ core-shell NCs compared to the bare UO_2_ core NCs. If uranyl cations were present in the UO_2_/CaF_2_ core-shell structure, one would expect greater deviation from the cubic structure in order to accommodate the U^VI^O_2_^2+^ moieties. Therefore, the greater cubic character in UO_2_/CaF_2_ suggests that extra uranyl cations are present in the reaction solution outside of the core-shell NCs. The exact reason for the presence of uranyl cations after shell growth is not yet understood and will be investigated by optimizing the synthesis conditions both for the UO_2_ core and UO_2_/CaF_2_ core-shell NCs.


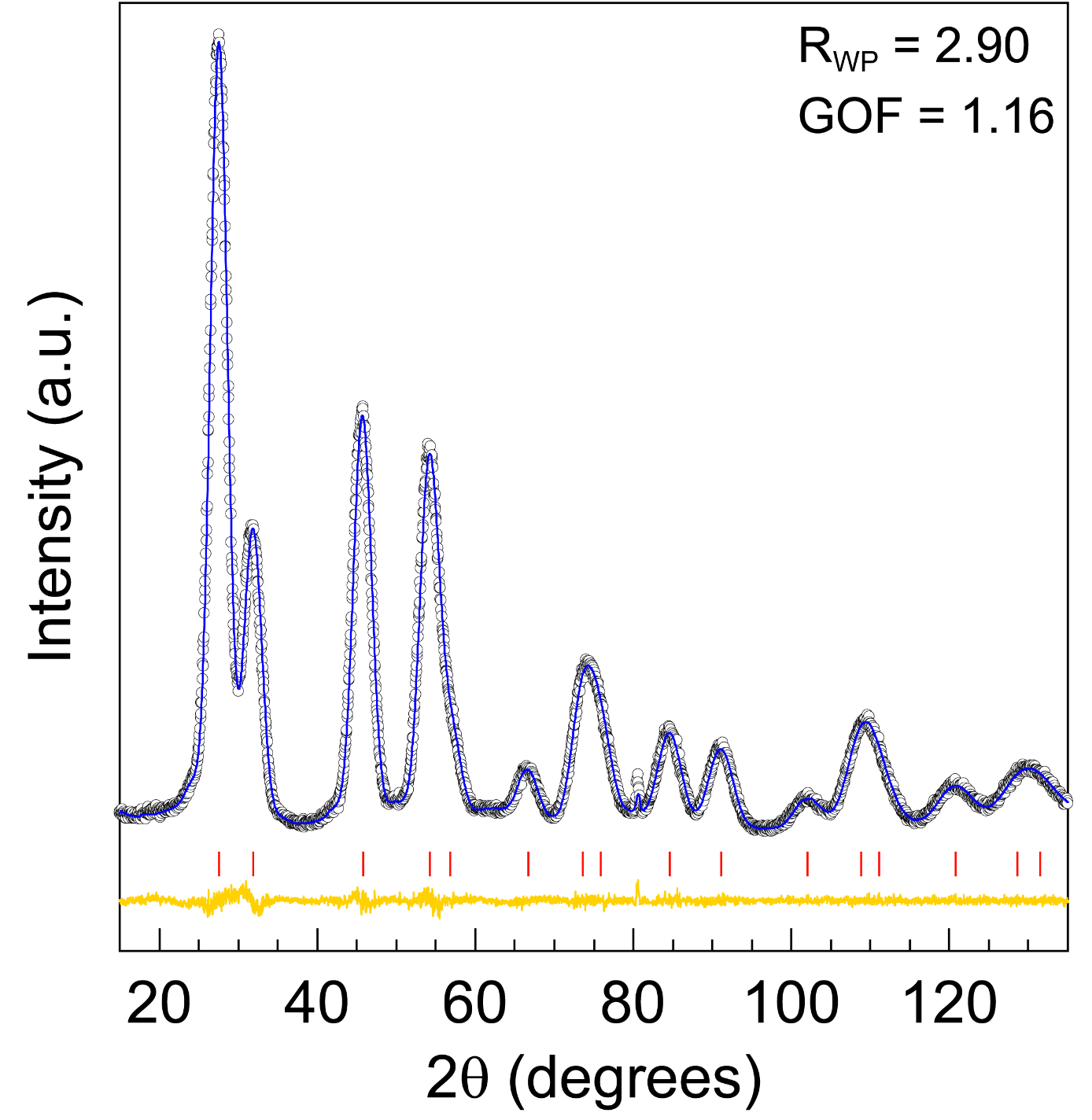


**Figure S24.** Experimental PXRD pattern (black open symbols) of ThO_2_ core NCs together with the corresponding Pawley refinement (blue solid line), difference curve (yellow solid line), and Bragg peaks’ positions (red vertical tick marks). The values of the weighted profile R factor (Rwp) and goodness-of-fit (GOF) are given to assess the quality of the refinement when combined with the visual inspection of the difference curve.





**Figure S25.** Low magnification HAADF-STEM image of ThO_2_/CaF_2_ core-shell NCs.

**Figure S26.** Experimental PXRD pattern (black open symbols) of ThO_2_/CaF_2_ core-shell NCs together with the corresponding Pawley refinement (blue solid line), difference curve (yellow solid line), and Bragg peaks’ positions (red and dark-red vertical tick marks). Due to the lattice mismatch between ThO_2_ and CaF_2_ (f = 2.5% for the microcrystalline materials), splitting of Bragg peaks starts to be visible and two phases where added in the refinement. The values of the weighted profile R factor (Rwp) and goodness-of-fit (GOF) are given to assess the quality of the refinement when combined with the visual inspection of the difference curve.

**5. Supplementary discussion related to the reduction of Ce(IV) into Ce(III) after CaF_2_ shell deposition**

The combined structural and spectroscopic data provide a coherent mechanistic interpretation of the unexpected reduction of Ce(IV) to Ce(III) during CaF_2_ shell growth. The starting CeO_2_ core NCs very likely contain a high concentration of surface oxygen-vacancy together with under-coordinated metal sites, as expected for *ca.* 7 nm NCs. In presence of calcium trifluoroacetate (*i.e.* molecular precursor used for CaF_2_ shell formation), such reactive surface sites readily interact with fluorinated species released during shell precursor decomposition. Thus, it is hypothesized that metal fluoride and/or oxyfluoride surface species locally form. Although the exact chemical composition of the latter is not accessible, EDX line scan analysis supports this interpretation as revealed by the existence of a 2-3 nm interface region where all cations (Ce, Ca) and anions (O, F) coexist with compositional gradients. The existence of fluoride and/or oxyfluoride motifs not only alter the chemical potentials but is also thermodynamically favorable for the appearance of Ce(III) species. Indeed, oxygen can be progressively removed from the near-surface region, generating oxygen vacancies that propagate inward while preserving the fluorite structure and in particular the cation network. Consequently, a topotactic vacancy-driven reduction reaction progressively converts Ce(IV) to Ce(III) and facilitates the transformation of the CeO_2_ core domain into a Ce(III)-rich, oxygen-deficient fluorite (*i.e.* CeO_2-x_) and/or sesquioxide-like phase (type-C Ce_2_O_3_). Importantly, such reduced fluorite derivatives or vacancy-ordered sesquioxide-type domains can retain an average cubic diffraction pattern as experimentally observed even though HR-XANES shows the complete reduction of Ce(IV) into Ce(III). Meanwhile, the outer region crystallizes as CaF_2_, and the *ca.* 2-3 nm region with cations/anions intermixing acts as a “buffer” interphase enabling both the topotactic transition of CeO_2_ and the CaF_2_ shell growth.

Although the available PXRD, HAADF-STEM imaging, EDX (chemical maps as well as line scan analysis), and HR-XANES measurements support this explanation, unambiguously resolving the atomic-scale arrangement of oxygen vacancies, fluorine incorporation, mixed Ce–Ca coordination, and possible defect superstructures will require a suite of complementary characterization techniques performed on several CeO_2_/CaF_2_ samples grown under different experimental conditions. Synchrotron-based PXRD (to reveal superstructure reflections if formation of a vacancy-ordered superstructure), EXAFS (to confirm whether Ce is predominantly coordinated to O or F to distinguish CeOF/Ce–F phases from pure oxide phases) and atomic-resolved EELS valence mapping (spatial localization of Ce(III) and Ce(IV)) are three characterization techniques that will be implemented in a future work.

**6. Supplementary references**

[1] D. Hudry, C. Apostolidis, O. Walter, T. Gouder, E. Courtois, C. Kübel, D. Meyer, *Chem. Eur. J.* **2012**, *18*, 8283.

[2] D. Hudry, C. Apostolidis, O. Walter, T. Gouder, E. Courtois, C. Kübel, D. Meyer, *Chem. Eur. J.* **2013**, *19*, 5297.

[3] S. Yang, L. Gao, *J. Am. Chem. Soc.* **2006**, *128*, 9330.

[4] F. Dang, K. Kato, H. Imai, S. Wada, H. Haneda, M. Kuwabara, *Cryst. Growth Des.* **2010**, *10*, 4537.

[5] F. Arteaga Cardona, N. Jain, R. Popescu, D. Busko, E. Madirov, B. A. Arús, D. Gerthsen, A. De Backer, S. Bals, O. T. Bruns, A. Chmyrov, S. Van Aert, B. S. Richards, D. Hudry, *Nature Commun.* **2023**, *14*, 4462.

[6] F. Arteaga Cardona, E. Madirov, R. Popescu, D. Wang, D. Busko, D. Ectors, C. Kübel, Y. M. Eggeler, B. A. Arús, A. Chmyrov, O. T. Bruns, B. S. Richards, D. Hudry, *ACS Nano* **2024**, *18*, 26233.

[7] D. Hudry, I. A. Howard, R. Popescu, D. Gerthsen, B. S. Richards, *Adv. Mater.* **2019**, *31*, 1900623.

[8] A. Morgenstern, O. Lebeda, J. Stursa, F. Bruchertseifer, R. Capote, J. McGinley, G. Rasmussen, M. Sin, B. Zielinska, C. Apostolidis, *Anal. Chem.* **2008**, *80*, 8763.
